# Art Therapy for Psychosocial Problems in Children and Adolescents: A Systematic Narrative Review on Art Therapeutic Means and Forms of Expression, Therapist Behavior, and Supposed Mechanisms of Change

**DOI:** 10.3389/fpsyg.2020.584685

**Published:** 2020-10-08

**Authors:** Liesbeth Bosgraaf, Marinus Spreen, Kim Pattiselanno, Susan van Hooren

**Affiliations:** ^1^Faculty of Healthcare and Social Work, NHL Stenden University of Applied Sciences, Leeuwarden, Netherlands; ^2^Alliade, Care Group, Heerenveen, Netherlands; ^3^KenVaK, Research Center for Arts Therapies, Heerlen, Netherlands; ^4^Faculty of Psychology, Open University, Heerlen, Netherlands; ^5^Faculty of Healthcare, Zuyd University of Applied Sciences, Heerlen, Netherlands

**Keywords:** art therapy, psychosocial problems, children, adolescents, systematic narrative review

## Abstract

**Background:** Art therapy (AT) is frequently offered to children and adolescents with psychosocial problems. AT is an experiential form of treatment in which the use of art materials, the process of creation in the presence and guidance of an art therapist, and the resulting artwork are assumed to contribute to the reduction of psychosocial problems. Although previous research reports positive effects, there is a lack of knowledge on which (combination of) art therapeutic components contribute to the reduction of psychosocial problems in children and adolescents.

**Method:** A systematic narrative review was conducted to give an overview of AT interventions for children and adolescents with psychosocial problems. Fourteen databases and four electronic journals up to January 2020 were systematically searched. The applied means and forms of expression, therapist behavior, supposed mechanisms of change, and effects were extracted and coded.

**Results:** Thirty-seven studies out of 1,299 studies met the inclusion criteria. This concerned 16 randomized controlled trials, eight controlled trials, and 13 single-group pre–post design studies. AT interventions for children and adolescents are characterized by a variety of materials/techniques, forms of structure such as giving topics or assignments, and the use of language. Three forms of therapist behavior were seen: non-directive, directive, and eclectic. All three forms of therapist behavior, in combination with a variety of means and forms of expression, showed significant effects on psychosocial problems.

**Conclusions:** The results showed that the use of means and forms of expression and therapist behavior is applied flexibly. This suggests the responsiveness of AT, in which means and forms of expression and therapist behavior are applied to respond to the client's needs and circumstances, thereby giving positive results for psychosocial outcomes. For future studies, presenting detailed information on the potential beneficial effects of used therapeutic perspectives, means, art techniques, and therapist behavior is recommended to get a better insight into (un)successful art therapeutic elements.

## Introduction

Psychosocial problems are highly prevalent among children and adolescents with an estimated prevalence of 10%−20% worldwide (Kieling et al., 2011; World Health Organization, 2018). These problems can severely interfere with everyday functioning (Bhosale et al., 2015; Veldman et al., 2015) and increase the risk of poorer performance at school (Veldman et al., 2015). The term psychosocial problems is used to emphasize the close connection between psychological aspects of the human experience and the wider social experience (Soliman et al., 2020) and cover a wide range of problems, namely, emotional, behavioral, and social. Emotional problems are often referred to as internalizing problems, such as anxiety, depressive feelings, withdrawn behavior, and psychosomatic complaints. Behavioral problems are often considered as externalizing problems, such as hyperactivity, aggressive behavior, and conduct problems. Social problems are problems related to the ability of the child to initiate and maintain social contacts and interactions with others. Often, emotional, behavioral, and social problems occur jointly (Vogels, 2008; Jaspers et al., 2012; Ogundele, 2018). The etiology of psychosocial problems is complex and varies with regard to the problem(s) and/or the specific individual. A number of theories seek to explain the etiology of psychosocial problems. The most common theory in Western psychology and psychiatry is the biopsychosocial theory, which assumes that a combination of genetic predisposition and environmental stressors triggers the onset of psychosocial problems (Lehman et al., 2017). But also, attachment theories get renewed attention (Duschinsky et al., 2015). These theories focus on the role of the early caregiver–child relationships and assume that (a lack of) security of attachment affects the child's self-(emotion)regulatory capacity and therefore his or her emotional, behavioral, and social competence (Veríssimo et al., 2014; Brumariu, 2015; Groh et al., 2016). Research has identified a number of biological, psychological, and environmental factors that contribute to the development or progression of psychosocial problems (Arango et al., 2018), namely, trauma, adverse childhood experiences, genetic predisposition, and temperament (Boursnell, 2011; Sellers et al., 2013; Wright and Simms, 2015; Patrick et al., 2019).

Psychosocial problems in children and adolescents are a considerable expense to society and an important reason for using health care. But, most of all, psychosocial problems can have a major impact on the future of the child's life (Smith and Smith, 2010). Effective interventions for children and adolescents, aiming at psychosocial problems, could prevent or reduce the likelihood of long-term impairment and, therefore, the burden of mental health disorders on individuals and their families and the costs to health systems and communities (Cho and Shin, 2013).

The most common treatments of psychosocial problems in children and adolescents include combinations of child- and family-focused psychological strategies, including cognitive behavioral therapy (CBT) and social communication enhancement techniques and parenting skills training (Ogundele, 2018). These interventions are designed with the idea that cognitions affect the way that children and adolescents feel and behave (Fenn and Byrne, 2013). However, this starting point is considered not suitable for all youngsters, in particular, for children and adolescents who may find it difficult to formulate or express their experiences and feelings (Scheeringa et al., 2007; Teel, 2007). For such situations in clinical practice, additional therapies are often offered. Art therapy (AT) is such a form of therapy.

AT is an experiential form of treatment and has a special position in the treatment of children and adolescents because it is an easily accessible and non-threatening form of treatment. Traditionally, AT is (among others) used to improve self-esteem and self-awareness, cultivate emotional resilience, enhance social skills, and reduce distress (American Art Therapy Association, 2017), and research has increasingly identified factors, such as emotion regulation (Gratz et al., 2012) and self-esteem (Baumeister et al., 2003) as mechanisms underlying multiple forms of psychosocial problems.

Art therapists work from different orientations and theories, such as psychodynamic; humanistic (phenomenological, gestalt, person-centered); psychoeducational (behavioral, cognitive–behavioral, developmental); systemic (family and group therapy); as well as integrative and eclectic approaches. But also, there are various variations in individual preference and orientation by art therapists (Van Lith, 2016). In AT, the art therapist may facilitate positive change in psychosocial problems through both engagement with the therapist and art materials in a playful and safe environment. Fundamental principles in AT for children and adolescents are that visual image-making is an important aspect of the natural learning process and that the children and adolescents, in the presence of the art therapist, can get in touch with feelings that otherwise cannot easily be expressed in words (Waller, 2006). The ability to express themselves and practice skills can give a sense of control and self-efficacy and promotes self-discovery. It, therefore, may provide a way for children and clinicians to address psychosocial problems in another way than other types of therapy (Dye, 2018).

Substantial clinical research concerning the mechanisms of change in AT is lacking (Gerge et al., 2019), although it is an emerging field (Carolan and Backos, 2017). AT supposed mechanisms of change can be divided into working mechanisms specific for AT and overall psychotherapeutic mechanisms of change, such as the therapeutic relationship between client and therapist or the expectations or hope (Cuijpers et al., 2019). Specific mechanisms of change for AT include, for instance, the assumption that art can be an effective system for the communication of implicit information (Gerge, and Pedersen, 2017) or that art-making consists of creation, observation, reflecting, and meaning-making, which leads to change and insight (Malchiodi, 2007).

Recently, it has been shown that AT results in beneficial outcomes for children and adolescents. Cohen-Yatziv and Regev (2019) published a review on AT for children and adolescents and found positive effects in children with trauma or medical conditions, in juvenile offenders, and in children in special education and with disabilities. While increasing insight into the effects of AT for different problem areas among children is collected, it remains unclear whether specific elements of AT interventions and mechanisms of change may be responsible for these effects. In clinical practice, art therapists base their therapy on rich experiential and intuitive knowledge. This knowledge is often implicit and difficult to verbalize, also known as tacit knowledge (Petri et al., 2020). Often, it is based on beliefs or common sense approaches, without a sound basis in empirical results (Haeyen et al., 2017). This intuitive knowledge and beliefs consist of (theoretical) principles, art therapeutic means and forms of expression, and therapist behavior [including interactions with the client(s) and handling of materials] that art therapists judge necessary to produce desired outcomes (Schweizer et al., 2014). Identifying the elements that support positive outcomes improves the interpretation and understanding of outcomes, provides clues which elements to use in clinical practice, and will give a sound base for initiating more empirical research on AT (Fixsen et al., 2005). The aim of this review is to provide an overview of the specific elements of art therapeutic interventions that were shown to be effective in reducing psychosocial problems in children and adolescents. In this review, we will focus on applied means and forms, therapist behavior, supposed mechanisms of change of art therapeutic interventions. As the research question was stated, i.e., which art therapeutic elements support positive outcomes in psychosocial problems of children and adolescents (4–20)?

## Methods

### Study Design

A systematic narrative review is performed according to the guidelines of the Cochrane Collaboration for study identification, selection, data extraction, and quality appraisal. Data analysis was performed, conforming narrative syntheses.

### Eligibility Criteria

In this review, we included peer-reviewed published randomized controlled trials (RCTs), non-randomized clinical controlled trials (CCTs), and studies with group pre–posttest designs for AT of psychosocial problems in children and adolescents (4–20 years). Studies were included regardless of whether AT was present within the experimental or control condition. Qualitative data were included when data analysis methods specific for this kind of data were used. Only publications in English, Dutch, or German were included. Furthermore, only studies in which AT was provided by a certified art therapist to individuals or groups, without limitations on duration and number of sessions, were inserted. Excluded were studies in which AT was structurally combined with another non-verbal therapy, for instance, music therapy. Studies on (sand)play therapy were also excluded. Concerning the outcome, studies needed to evaluate AT interventions on psychosocial problems. Psychosocial problems were broadly defined as emotional, behavioral, and social problems. Considered emotional (internalizing) problems were, for instance, anxiety, withdrawal, depressive feelings, psychosomatic complaints, and posttraumatic stress problems/disorder. Externalizing problems were, for instance, aggressiveness, restlessness, delinquency, and attention/hyperactivity problems. Social problems were problems that the child has in making and maintaining contact with others. Also included were studies that evaluated AT interventions targeted at children/adolescents with psychosocial problems and showed results on supposed underlying mechanisms such as, for instance, self-esteem and emotion regulation.

### Searches

Fourteen databases and four electronic journals were searched: PUBMED, Embase (Ovid), PsycINFO (EBSCO), The Cochrane Library (Cochrane Database of Systematic Reviews, Cochrane Central Register of Controlled Trials), Web of Science, Cinahl, Embase, Eric, Academic Search Premier, Google Scholar, Merkurstab, ArtheData, Relief, and Tijdschrift Voor Vaktherapie (Journal of Arts Therapies in the Netherlands). A search strategy was developed using keywords (art therapy in combination with a variety of terms regarding psychosocial problems) for the electronic databases according to their specific subject headings or structure. For each database, search terms were adapted according to the search capabilities of that database (Appendix 1). The search period had no limitation until the actual first search date: October 5, 2018. The search was repeated on January 30, 2020. If online versions of articles could not be traced, the authors were contacted with a request to send the article to the first author. The reference lists of systematic reviews, found in the search, were hand searched for supplementing titles to ensure that all possible eligible studies would be detected.

### Study Selection

A single RefWorks file of all identified references was produced. Duplicates were removed. The following selection procedure was independent of each other carried out by four researchers (LB, SvH, MS, and KP). Titles and abstracts were screened for eligibility by three researchers (LB, SvH, and KP). The full texts were subsequently assessed by three researchers (LB, MS, and KP) according to the eligibility criteria. Any disagreement in study selection between a pair of reviewers was resolved through discussion or by consultation of the fourth reviewer (SvH).

### Quality of the Studies

The quality of the studies was assessed by two researchers (LB and KP) applying the EPHPP “Quality Assessment Tool for Quantitative Studies” (Thomas et al., 2004). Independent of each other, they came to an opinion, after which consultation took place to reach an agreement. To assess the quality, the Quality Assessment Tool was used, which has eight categories: selection bias, study design, confounders, blinding, data collection methods, withdrawal and dropouts, intervention integrity, and analysis. Once the assessment was completed, each examined study received a mark ranging between “strong,” “moderate,” and “weak.” The EPHPP tool has a solid methodological rating (Thomas et al., 2004).

### Data Collection and Analysis

The following data were collected from the included studies: continent/country, type of publication of study, year of publication, language, impact factor of the journal published, study design, the primary outcome, measures, setting, type of clients, comorbidity, physical problems, total N, experimental N, control N, proportion male, mean age, age range, the content of the intervention, content control, co-intervention, theoretical framework AT, other theoretical frameworks, number of sessions, frequency sessions, length sessions, outcome domains and outcome measures, time points, outcomes, and statistics. An inductive content analysis (Erlingsson and Brysiewicz, 2017) was conducted on the characteristics of the employed ATs concerning the means and forms of expression, the associated therapist behavior, the described mechanisms of change, and whether there were significant effects of the AT interventions. A narrative analysis was performed.

## Results

### Study Selection

**Figure 1:**
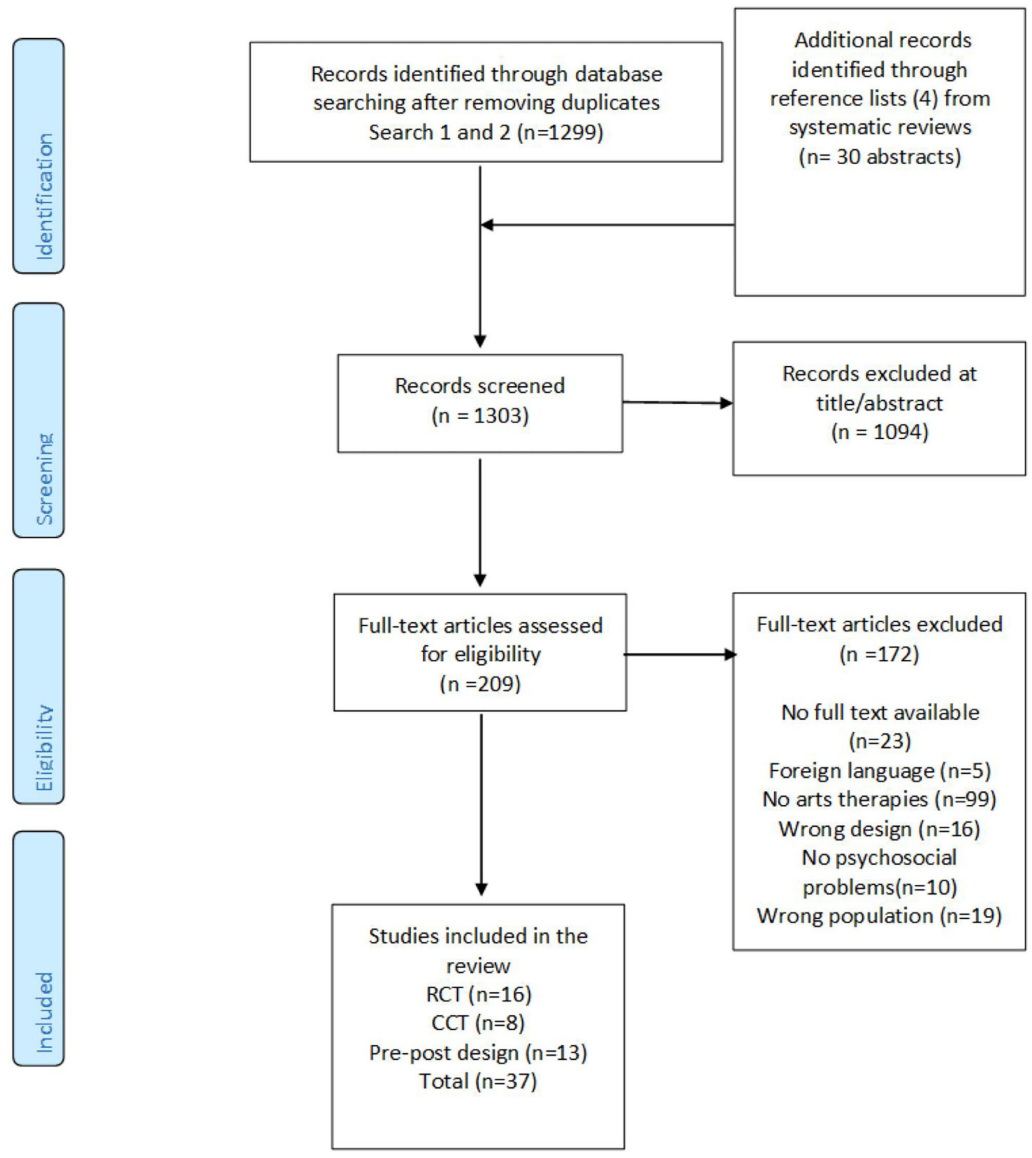
PRISMA (Preferred Reporting Items for Systematic Reviews and Meta-Analyses) flow chart.

The first search (October 2018) yielded 1,285 unique studies. In January 2020, the search was repeated, resulting in 14 additional unique studies, making a total of 1,299. Four additional studies identified from manually searching the reference lists from 30 reviews were added, making a total of 1,303 studies screened on title and abstract. In the first search, 1,085 studies, and in the second search, nine studies were excluded, making a total of 1,094 studies being excluded on title and abstract. This resulted in 209 full-text articles to assess eligibility. In the full-text selection phase, from the first search, another 167 studies were excluded; in the second search, five studies were excluded. This makes a total of 172 studies being excluded in the full-text phase. Twenty-three studies were excluded because a full text was unavailable; five studies because the language was not English, Dutch, or German; 99 studies did not meet the AT definition; 16 studies had a wrong design; 10 studies did not treat psychosocial problems; and 19 studies concerned a wrong population. In total, 37 studies were included (see Figure 1 for an overview of the complete selection process).

### Study Design

The final review included 16 RCTs, eight CCTs, and 13 single-group pre–post designs (total *n* = 37). Of the RCTs, a mixed-method design, involving both quantitative and qualitative data, was used in two studies. In one RCT, the control group received AT meeting our criteria, while the experimental group did not receive such a therapy (11). In another RCT, the experimental and the control group both received AT meeting our criteria (13). Also, two CCT studies used a mixed-method design, but these qualitative results were not included due to inappropriate analysis. Of the single-group pre–posttest designs, two studies had a mixed-method (quantitative and qualitative) design (Table 1).

**Table 1:** Study characteristics/outcome.

**References**	**Design/Time points**	**Quality assess-ment rate**	**Study population**	**Number of participants (treated/control)**	**Type (group or individual or both), frequency, duration**	**Control intervention/sare as usual**	**Outcome domain/Measure**	**Results**	**Qualitative results**
Bazargan and Pakdaman (2016)	RCT, pre–posttest	Strong	Age: 14–18 with internalizing and externalizing problems	60 (30/30)	Group, six sessions, 60 min	Not described	Achenbach System of Empirically Based Assessment (ASEBA) (2001): internalizing and externalizing problems	Art therapy significantly reduced internalizing problems; effect in reducing externalizing problems was not significant.	-
Beebe et al. (2010)	RCT, pre–posttest, follow-up: 6 months	Weak	Age: 7–14 with persistent asthma	22 (11/11)	Group, seven sessions, 60 min, once a week	Care as usual	Beck Youth Inventories Second Edition: self-reported adaptive and maladaptive behaviors and emotions; the Pediatric Quality of Life (Peds QL) Asthma Module and the Peds QL Asthma Module Parent Report for Children: impact of asthma on the quality of life; Draw a Person Picking an Apple from a Tree: evaluation part from the Formal Elements of Art Therapy Scale (FEATS): coping abilities and resourcefulness	Statistically improved Beck anxiety and self-concept scores from the child-reported Beck Inventories. Disruptive behavior, anger, and depression did not change statistically. Improved problem-solving and affect drawing scores on the FEATS. Statistically improved parent and child worry, communication, and parent and child total quality of life scores. At 6 months, the active group maintained (affect drawing scores, worry, and quality of life); Bec[6mm]k Anxiety score Frequency of asthma exacerbations did not differ between the two groups.	-
Beh-Pajooh et al. (2018)	RCT, pre–posttest	Moderate	Mean age:12, male children with ID and externalizing behaviors	60 (30/30)	Group, 12 sessions two times a week, 45 min	The control group did not receive any intervention program	Conditional Reasoning Problems: externalizing behaviors; Bender Visual-Motor Gestalt Test (BVMGT): emotional problems	The mean levels of externalizing behaviors between the intervention group and the control group were significantly different. No significance for emotional problems.	-
Chapman et al. (2001)	RCT, measures at 1-week, 1-month, and 6-months intervals	Moderate	Age: 7–17, mean age: 10.7, 70.6% male admitted to a Level I trauma center for traumatic injuries	58 (31/27)	Individual, once	Care as usual, including child life services, art therapy, social work and psychiatric consults	PTSD-I: self-report measure that asks the individuals to respond to a 20-item inventory of symptoms based primarily on the diagnostic PTSD criteria in the DSM-IV	No statistically significant differences in the reduction of PTSD symptoms between the experimental and control groups. Children receiving the art therapy intervention showed a reduction in acute stress symptoms but not significantly.	-
Freilich and Shechtman (2010)	RCT, baseline, after, follow-up (3 months later) Process measures five times throughout the intervention Critical incidents: following each session	Moderate	Age: 7–15, learning disabilities, 70 % male	93 (42/51)	Group, 22 weeks, 60 min	Three hours of teaching	Child Behavior Checklist (CBCL)/The Teacher Evaluation Form (TRF): adjustment; Working Alliance Inventory: bonding with group members	Significant reduction in internalizing and externalizing problems. Control group scored higher on process variables (bonding and impression of therapy); bonding was associated with outcomes only in the therapy condition (not significant).	-
Hashemian and Jarahi (2014)	RCT, pre–posttest	Moderate	Age: 8–15, educable ID students, IQ 50 to 70, boys and girls	20 (10/10)	Group, 12 sessions, 75 min 2 times a week within 2 months	Care as usual: routine education and activity of their programs in school	Rutter Behavior Questions (form for teachers): aggression, hyperactivity, social conflict, antisocial behaviors, attention deficits; Good enough draw a person test: aggression behavior	Painting therapy was effective. The mean scores of aggression in the intervention group and the control group were significantly different.	-
Kymissis et al. (1996)	RCT, pre–posttest	Moderate	Age: 13–17, variety of diagnoses: Conduct Disorder (CD), Oppositional Defiant Disorder (ODD), Depressive Disorder, Bipolar Disorder and Borderline Personality Disorder (BPD)	37 (18/19)	Group, eight sessions, four sessions per week for 2 weeks	Discussion group with same co-therapists as treatment group: free discussion with minimal directions	Children's Global Assessment Scale (CGAS): general functioning; Inventory of Interpersonal Problems (IIP): distress from interpersonal sources	Both groups significant improvement in general functioning, SCIT members the highest degree (however not significant). No significance in either group on interpersonal variables of Assertiveness, Sociability, or Responsibility.	-
Liu (2017)	RCT, pre–posttest	Strong	Age: 6–13, one or more traumatic experiences and self-reported or parent-reported sleep-related problems	41 (21/20)	Group, eight sessions of 50 min in 2 weeks	Care as usual: counseling/ medications and same group activities except the experimental SF-AT treatment, regular group activities: art, music, sports, computer games and dance	The Connecticut Trauma Screen (CTS) and Child Reaction to Traumatic Events Scale-Revised (CRTES): PTSD; Sleep Self-Report (SSR): sleep (trauma-related)	Findings indicated that the SF-AT significantly alleviated PTSD and sleep symptoms.	-
Lyshak-Stelzer et al. (2007)	RCT, pre–posttest	Weak	Age: 13–17, 55,2% male, chronic child post-traumatic stress disorder (PTSD)	29 (14/15)	Individual approach in group, 16 sessions once a week	Care as usual: standard arts- and craft-making activity group	The UCLA PTSD Reaction Index for DSM-IV Child Version: PTSD symptoms in children ages 7–12	Significant treatment-by-condition interaction indicating the TF-ART condition had a greater reduction in PTSD symptoms.	-
Ramin et al. (2014)	RCT, pre–posttest	Moderate	Age: 7–11, intense aggressive behaviors, boys and girls	30 (15/15)	Group, 10-week intervention, participants had the choice of attending weekly 2-h art therapy sessions, a minimum of 7 sessions were included in the study	Not described	Children's Inventory of anger (ChIA): anger; Coppersmith Self-esteem Inventory: self-esteem	The art therapy group showed a significant reduction of anger and significant improvement of self-esteem compared with the control group. The educational self-esteem subscale did not show a significant reduction in comparison with the control group.	-
Regev and Guttmann (2005)	RCT, pre–posttest, four groups: 1 experimental, three control	Moderate	Age: 8–13, Male: 63.2%, primary-school children with learning disorders	104 (25/25/29/25)	Group, 25 weeks, 45 min	Three control groups: control group A (games group) various in-class games, control group B (art therapy group) art projects in an art-therapy fashion by art therapist, group C: no intervention	LSDQ The Loneliness and Social Dissatisfaction Questionnaire: socially lonely and dissatisfaction; CSCS The Piers-Harris Children's Self-Concept Scale: self-esteem; IARQ The Intellectual Achievement Responsibility Questionnaire: responsibility for successes/failures at school; CS The Children's Sense of Coherence Scale: a sense of empowerment	Children in the art therapy group did not score better than those in any other group on any of the dependent variables.	-
Richard et al. (2015)	RCT, pre–posttest	Moderate	Age: 8–14, ASD (Autism Spectrum Disorder)	19 (10/9)	Individual, once 60 min	Magneatos, a three-dimensional construction set for building three-dimensional designs	The Diagnostic Analysis of Non-verbal Accuracy 2-Child Facial Expressions (DANVA 2-CF): measuring the accurate sending and receiving of non-verbal social information	No significant difference between the treatment and control group on the accurate sending and receiving of non-verbal social information; however, the treatment group had more considerable improvement than the control.	-
Rosal (1993)	RCT, pre–posttest, mixed-method	Moderate	Mean age: 10.2, moderate to severe behavior problems	36 (12/12/12)	Group, 20 times, two times a week, 50 min	Cognitive-behavioral art therapy	The TRS: problem behavior; The Children's Nowicki-Strickland Internal-External Locus of Control (CNS-IE): locus of control; a personal construct drawing interview (PCDI) (qualitative); two case examples are described	No significant results for LOC. Both cognitive-behavioral art therapy and the art as therapy group showed significant results for problem behavior, although art as therapy marginal.	Two children improved in LOC and behavior (case examples)
Schreier et al. (2005)	RCT, pre–posttest within 24 h of hospital admission, repeated at 1 month, 6 months, and 18 months	Moderate	Mean age: 10, children hospitalized for a minimum of 24 h after physical trauma	57 (27/30)	Individual, once for approximately an hour	Care as usual: standard hospital services	UCLA Posttraumatic Stress Disorder Reaction Index (UCLA PTSD-RI): PTSD	The art therapy intervention showed no sustained effects on the reduction of PTSD symptoms.	
Siegel et al. (2016)	RCT, pre–posttest, mixed-method	Moderate	Age: 4–16, mean age: 8.3, pediatric patients with a wide range of serious medical diagnoses	25 (13/12)	Individual, once 90 min	Control group: the same assessments as the treatment group but did not receive therapy until after all of the assessments were collected	Question asked: how are you feeling right now about your stay in the hospital? Children could choose a series of faces expressing emotions (mood) Complemented at posttest with: a qualitative interview with two questions: Is there anything you want to tell us about how are you feeling right now?	No significant improvements in mood for children following therapy sessions. Compared to the children in the wait-list control group, there was a trend of improvement in mood reported by the children immediately following the therapy.	-
Tibbetts and Stone (1990)	RCT, pre–posttest	Strong	Mean age: 14.6, seriously emotionally disturbed (SED)	16 (8/8)	Group, once a week, 6 weeks, 45 min	Weekly socialization sessions by the same professional with individual sessions lasting 45 min, activities: playing board games, talking about weekend activities, taking walks on the school grounds	The Burks Behavior Rating Scales (BBRS): behavioral and emotional functioning; the Roberts Apperception Test (RATC): personality	Overall, no significant differences were found on the BBRS, but both groups demonstrated overall positive changes across almost all measured categories of behavioral and emotional functioning. The experimental group demonstrated statistically significant improvement in attention span and sense of identity (BBRS). RATC: significant improvement overall. The experimental group demonstrated significant score reductions in Reliance Upon Others, degree of perceived support available from others in the environment (Support/Other), and their positive expressed feelings about themselves (Support/Child). At the same time, significant reductions were also found in levels of Depression, Rejection, and Anxiety.	-
Jang and Choi (2012)	CCT, pre–posttest, follow-up after 3 months	Weak	Age: 13–15, boys and girls in an educational welfare program needing emotional and psychological help	16 (8/8)	Group, 18 times, weekly 80 min	Not described	Shin's (2004) ego resilience scale: ego resilience	A significant increase in ego resilience between pre-, post-, and follow-up. There was a positive effect on the regulation and release of emotions (not significant).	-
Khadar et al. (2013)	CCT, pre–posttest, 1 month follow-up	Weak	Age: 7–11, boys with symptoms of separation anxiety disorder	30 (15/15)	Group, 12 times twice a week, 40 min	Not described	The Child Symptom Inventory-4 (CSI-4): emotional and behavioral disorders	The experimental group had a significant decrease in the symptoms of Separation Anxiety Disorder, while the control group showed no significant difference.	-
Khodabakhshi Koolaee et al. (2016)	CCT, pre–posttest, follow-up after 1 month	Weak	Age: 8–12, boys and girls with leukemia cancer who had one score above the mean scores of anxiety and anger	30 (15/15)	Group, 11 sessions, twice a week, 60 min	The control group did not receive any intervention	Spence Children's Anxiety Scale: anxiety; Children's Inventory of Anger (ChIA): anger	A significant difference between the pretest and post-test scores in aggression and anxiety.	-
Pretorius and Pfeifer (2010)	CCT, pre–posttest control group design and posttest only control group design	Weak	Age: 8–11, girls with a history of sexual abuse	25 (6/6/6/7)	Group, eight times	The control group did not receive any intervention	The Trauma Symptom Checklist for Children (TSCC): depression, anxiety, and sexual trauma; The Human Figure Drawing (HFD): self-esteem	The experimental groups improved significantly compared to the control groups concerning anxiety and depression. No significance in sexual trauma and low self-esteem.	-
Ramirez (2013)	CCT, pre–posttest, mixed-method	Weak	Male high school freshmen students living in poverty	156 Exp.: 80 (29/26/25) Contr.: 76 (24/26/26)	Group, 12 sessions once a week	Academic work	The Behavior Assessment System for Children Second Edition (BASC-2): behavioral and emotional problems; qualitative questionnaire for responses to open-ended prompts	Three groups: (1) Honors track: art therapy group improved significantly on inattention/hyperactivity more than those in the control group, but not on anxiety, depression, self-esteem, internalizing problems, emotional symptoms, and personal adjustment; (2) Average track: personal adjustment and self-esteem improved significantly more for art therapy participants than for those in the control group, but not on anxiety, depression, inattention/hyperactivity problems, internalizing problems and emotional symptoms. No statistically significant differences were found for participants in the (3) At-risk track.	Participant responses: through the creative process, peer interactions increased, ventilation of uncomfortable feelings occurred, and outlets for alleviating stress were provided.
Steiert (2015)	CCT, design with control sample	Moderate	Age: 10–16, seven with a life-threatening illness and two brothers/sisters	Exp.: 9 (control sample 780)	Individual, six sessions, 90 min, varying from one to three times a week	Control sample Feel K-J	Feel-KJ: emotion regulation	Significant deviations from the control group for emotion regulation. From a sample of nine participants, two children differed significantly, and five children very significantly from the value of the standard sample. Maladaptive strategies: highly significant for three of the children.	-
Wallace et al. (2014)	CCT, 1 week after the procedure, 1 month post, 3 months post	Strong	Age: 6–18, siblings of pediatric patients who had undergone pediatric hematopoietic stem cell transplant	30 (20/10)	Individual, three times, session's duration varied from 90 min to 2 h	No treatment	The Revised Children's Manifest Anxiety Scale Second Edition (RCMAS−2: anxiety; Second Edition UCLA PTSD Index for DSM-IV: PTSS; the Piers-Harris Children's Self-Concept Scale: self-concept	Compared to the control group, the intervention group showed significantly lower levels of posttraumatic stress symptoms at the final session. Improvements in sibling psychosocial functioning associated with participation in the art therapy interventions. No intervention vs. control group difference for self-concept and anxiety.	-
Walsh (1993)	CCT, pre–posttest time-series design, follow up after a month, mixed-method	Strong	Age: 13–17, hospitalized suicidal boys and girls	39 (21/18)	Group, two times 90 min	Three hours of informal recreational activities (gymnasium free time)	Beck Depression Inventory (BDI): depression; the Coopersmith Self-Esteem Inventory (SEI): self-esteem	Both groups improved on all measures during and after hospitalization but not significantly.	-
Chaves (2011)	Single group pre–posttest, mixed-method	Moderate	Age: 12–20, eating disorder patients, one boy, seven girls	8	Group, four times once a week, 240 min	No control	The Subjective Units of Distress (SUDS) scale and visual analog scale (VAS): four negative mood states commonly found in individuals with eating disorders; the Rosenberg Self-Esteem Scale and the Hartz Art Therapy Self-Esteem Scale: global self-esteem and art therapy-related self-esteem	Global self-esteem did not change. Self-esteem related to art therapy trended upward, though still did not show significant change. The SUDS (distress) and VAS (negative mood) showed the most considerable change after the first group session, but not significantly.	-
D'Amico and Lalonde (2017)	Single group pre–posttest, mixed-method	Weak	Age:10–12, 5 boys and one girl with ASD (Autism Spectrum Disorder) who required varying degrees of substantial support	6	Group, once a week for 21 weeks, 75 min	No control	The Parent and Student Forms of the Social Skills Improvement System Rating Scales (SSIS–RS): social skills and problem behaviors; Observations of the children's progress recorded by the art therapists in their clinical notes (qualitative)	Significant reduction of hyperactivity/inattention. No significant changes in mean, standard scores for social skills. No statistically significant mean changes in the standard scores for problem behaviors. Art therapy enhanced the ability of children with ASD (Autism Spectrum Disorder) to engage and assert themselves in their social interactions, while reducing hyperactivity and inattention.	The children demonstrated a shift in self-image, were more confident and assured of their skills. Capable of expressing their ideas, thoughts, and feelings and sharing these. The children enjoyed providing and receiving feedback about their artwork. They appeared to initiate social exchanges independently. Increased capacity to reflect on their behaviors and display self-awareness.
Devidas and Mendonca (2017)	Single group pre–posttest	Weak	47.61% age: 11–12, 9.52% age: 13–14, orphans with low self-esteem	42	Group, 10 times once a week	No control	Rosenberg Self Esteem Scale: self-esteem	Art therapy was significantly effective in improving the level of self-esteem.	-
Epp (2008)	Single group pre–posttest	Moderate	Age: 6–12, students on the autism spectrum	66	Group, once a week 60 min	No control	The SSRS: social behavior problems	Significant improvement in assertion scores, internalizing behaviors, hyperactivity scores, and problem behavior scores. No significant change for responsibility.	-
Hartz and Thick (2005)	Two intervention group pre–posttest design	Moderate	Age: 13–18, female juvenile offenders	27 (12/15)	Group, 10 times during 12 weeks, 90 min	No control	The Harter Self-Perception Profile for Adolescents (SPPA): self- esteem; The Hartz Art Therapy Self Esteem Questionnaire (Hartz AT-SEQ): development of mastery, social connection, and self-approval	No significant differences on the Hartz AT-SEQ: self-esteem. Both groups (a/b) reported increased feelings of mastery, connection, and self-approval (not significant). The art psychotherapy (b) group showed a significant increase in domains of close friendship and behavioral conduct, whereas the art as therapy group (a) did in the domain of social acceptance.	-
Higenbottam (2004)	Single group pre–posttest	Weak	Age: 13–14, eighth-grade students, reasons for referral varied: eating disorders, suspected eating disorders, substance abuse, low self-esteem, negative body image, and relational aggression	7	Group, eight times once a week, 90 min	No control	Questionnaires: student's feelings around body image and self-esteem adapted from Daley and Lecroy's Go Grrrls Questionnaire	Significant improvements in body image and self-esteem. Participation in the art therapy group may significantly contribute to improved body image and self-esteem and hence the academic and psychological adjustment of adolescent girls.	-
Jo et al. (2018)	Single group pre–posttest	Moderate	Age: 7–10, siblings of children with cancer, boys and girls	17	Group, 12 times once a week, 60 min	No control	Revised Children's Manifest Anxiety Scale, adapted by Choi and Cho to fit a Korean context: anxiety; DAS test by Silver: Depression; K-CBCL standardized into Korean from the original CBCL: problem behavior; self-esteem: scale developed by Choi and Chun	Significant improvement for self-esteem. Significant decrease in somatic symptoms, aggressiveness, externalizing problems, total behavior problem scale, and emotional instability. No significant results for withdrawal, anxiety/depression, social immaturity, thought problems, and attention problems.	-
Pifalo (2002)	Single group pre–posttest	Moderate	Age: 8–17, girls victims of sexual abuse	13	Group, 10 times once a week, 90 min	No control	The Briere Trauma Symptom Checklist for Children (TSCC): trauma symptoms	Significantly reduced anxiety, post-traumatic stress, and dissociative symptomatology scores. Participants showed a decrease in depression, anger, and sexual concerns, although these decreases were not large enough to be statistically significant.	-
Pifalo (2006)	Single group pre–posttest	Moderate	Age: 8–10, 11–13, and 14–16, children with histories of sexual abuse	41	Group, eight times once a week, 60 min	No control	The Briere Trauma Symptom Checklist for Children (TSCC): trauma symptoms	A statistically significant reduction of Anxiety, Depression, Anger, Posttraumatic Stress, Dissociation, Dissociation-Overt, Sexual Concerns, Sexual Preoccupation, and Sexual Distress. No significant change for Hyper-response, Dissociation-Fantasy.	-
Rowe et al. (2016)	Pre–posttest, mixed-method	Moderate	Age:11–20, refugees	30	60% individual, 40 % group	No control	Hopkins Symptoms Checklist (HSC): symptoms of anxiety and depression; The Strengths and Difficulties Questionnaire (SDQ): behavior and performance in school; Harvard Trauma Questionnaire (HTQ): previous experience of trauma; Piers-Harris Self-Concept Scale (PHSCS): self-concept	Improvements in anxiety and self-concept but not significantly.	-
Saunders and Saunders (2000)	Pre–posttest, multiyear evaluation	Weak	Age: 2–16, problems: hyperactivity, poor concentration, poor communication, defiant behavior, lying/blaming, poor motivation, change in sleep routine, manipulation and fighting	94	Individual, between 2 and 96 sessions	No control	Rating on 24 behaviors typically identified as symptomatic of individual and family dysfunction	Significant positive impact on the lives of clients/families. Clients showed a significant decrease in frequency and severity ratings of problematic behaviors.	-
Sitzer and Stockwell (2015)	Single group pre–posttest within-subjects, mixed-method	Weak	Elementary school students with a variety of concerns: emotional dysregulation, lack of social skills, depression, anxiety, lack of focus and concentration, many a history of trauma	43	Group, 14 sessions, once a week 60 min	No control	The Wellness Inventory: school functioning attributes: emotional, behavioral, cognitive, and social problems and resilience. Teachers observed students throughout the day for relevant changes in mood and behavior (qualitative)	Results indicated significant increases in resilience, social and emotional functioning. No significant change for behavioral problems.	Overall functioning improves. Improvements in emotional expression, cognition, behavioral interaction, and resilience.
Stafstrom et al. (2012)	Pre–posttest	Moderate	Age: 7–18, epilepsy (any type) for at least 6 months	17	Group, four sessions 90 min	No control	Childhood Attitude Toward Illness Scale	No significant change in pre- vs. post-group CATIS scores.	-

*ASD, Autism Spectrum Disorder; CATIS, Childhood Attitude Toward Illness Scale; CBCL, Child Behavior Checklist; CCT, Clinical Controlled Trial; DAS, Draw a Story; DSM, Diagnostic Statistic Manuel of Mental Disorders; ID, Intellectual Disability; RCT, Randomized Controlled Trial; LOC, Locus of Control; PTSD, Post Traumatic Stress Disorder; SCIT, Synallactic Collective Image Therapy; SF-AT, Solution Focused Art Therapy; SSRS, Social Skills Rating System; TF-ART, Trauma Focused Expressive Art Therapy; TRS, Teacher Rating Scales; UCLA, University of California at Los Angeles Post-traumatic Stress Disorder Reaction Index*.

### Quality of the Studies

Of the 16 RCTs, two studies were evaluated as weak, 11 studies received a moderate score, and three studies were labeled as strong. Concerning the CCTs, five studies were evaluated as weak, one study as moderate, and two studies as strong. Of the 13 pre–posttest designs, five studies were assessed as weak and eight studies as moderate (Table 1).

### Study Population

The studies in this review included children and adolescents (ages 2–20) with a wide range of psychosocial problems and diagnoses. Most of the studies included children from the age of 6 years onward, with children's groups ranging from 6 to 15, adolescent groups ranging from 11 to 20, and mixed groups with an age range of 6–20 years. In 13 studies, both boys and girls were included, three studies only included boys, three studies only included girls, and 18 studies did not report the gender of the participants. Psychiatric diagnoses were reported, such as depression, autism spectrum disorder (ASD), conduct disorder (CD), post-traumatic stress disorder (PTSD), and mild intellectual disability (MID). However, also more specific problems were reported, such as children with suicidal thoughts and behavior, children having a brother/sister with a life-threatening disease, boys and girls in an educational welfare program needing emotional and psychological help, and orphans with a low self-esteem. Another group of children that were reported had medical concerns, such as persistent asthma, traumatic injuries, or serious medical diagnoses such as cancer, often combined with anxiety problems and/or trauma-related problems (Table 1).

### Number of Participants

The sample sizes of the RCTs ranged from 16 to 109. The total number of children of all RCTs was 707, of which 317 were allocated to an experimental condition and 390 to a control condition (Table 1). The sample sizes of the CCTs ranged from 15 to 780, and the total number of participants was 1,115. The total number of participants who received an AT treatment was 186; the total number of the control groups was 929. Notice that the sample size for the CCTs was influenced by one study in which a control sample database of 780 was used. The sample size of the included pre–posttest designs ranged from 8 to 94 participants, with a total number of 411 participants (Table 1).

### Type of Intervention, Frequency, and Treatment Duration

In the 37 studies, a total of 39 AT interventions were studied. In two studies, two AT interventions were studied. Of the 39 interventions, 30 studies evaluated group interventions, seven studies evaluated an individually offered intervention, one study evaluated an individual approach within a group setting, and in one study, the intervention was alternately offered as a group intervention or as an individual intervention. The number of sessions of the AT interventions varied from once to 25 times. The frequency of the AT interventions varied from once a week (*n* = 14) or twice a week (*n* = 5) and variations such as four times a week in 2 weeks (*n* = 1); six sessions were varying from one to three times a week (*n* = 1), 10 sessions during 12 weeks (*n* = 1), and eight sessions in 2 weeks (*n* = 1). The frequency of sessions has not been reported in nine studies. In five studies, the intervention was offered once (Table 1).

### Control Interventions

In six RCTs, care, as usual, was given to the control groups. In study four, this also concerned AT, but it was offered in a program that consisted of different forms of treatment as child life services, social work, and psychiatric consults and therefore did not meet our criteria for inclusion. The control groups receiving “care as usual” received routine education and activities of their programs in school (6); counseling/medications and group activities as art, music, sports, computer games, and dance (8); standard arts- and craft-making activities in a group (9); and standard hospital services (14). One study did not specify what happened as care as usual (2). In five RCTs, a specific intervention of activity was offered in the control condition. These control interventions involved 3 h of teaching (5), a discussion group (7), offering play material (magneatos) (12), and a range of games (11), and one study offered weekly socialization sessions, these sessions were offered by the same professionals as the experimental group, and activities were playing board games, talking about weekend activities, and taking walks on the school grounds (16). Two RCT studies did not mention the condition in the control group (1, 10). Two studies mentioned that the control group did not receive any intervention program (3, 11). One study mentioned that the control group had the same assessments as the treatment group but did not receive therapy until all of the assessments were collected (15).

Regarding the eight CCTs, two studies described the control condition in more detail, consisting of academic work (21) or 3 h of informal recreational activities (24). No intervention was offered to the control group in four studies (19, 20, 22, 23). The control intervention was not described within two studies (17, 18) (Table 1).

### Applied Means and Forms of Expression

The applied means and forms of expression in the AT interventions could be classified into three categories: *art materials/techniques, topics/assignments* given, and *language as a form of verbal expression* accompanying the use of art materials. Results will be shown for 39 AT interventions in total, coming from 37 studies (Table 2). Two studies applied two different types of AT interventions. These two types of AT will be referred to as 13 a/b and 29 a/b.

**Table 2:** Characteristics AT interventions.

**References**	**Applied means and forms of expression**	**Art materials**	**Therapist behavior**	**Supposed mechanism(s) of change**
Bazargan and Pakdaman (2016)	Painting sessions. Subjects had 45 min to 1 h to draw. In the end, subjects had 15 min to talk with the therapist and other members about works, feelings, interests, and events. Topics: warm-up activities using painting and coloring, learning about art media, general topics, first childhood memory/family relations, and the directed mental image, visualization, dream and meditation, anger releasing.	Cardboard and acrylic paint and drawing materials	No information given	Reveal what they have inside; leads to new activities and enhances experiences; provides an individual with opportunities through which they can freely express their feelings, affections, needs, and knowledge; achieving a feeling of security toward unpleasant memories of a traumatic event; emotions and thoughts are influenced by conflicts, fears, and desires, and painting allows patients to express them symbolically; offering opportunities to regain a sense of personal agency; explore existential concerns; reconnect to the physical body
Beebe et al. (2010)	Opening activity, discussing the weekly topic and art intervention related to chronic illness, art-making, opportunity for the patients to share their feelings related to the art they created, and closing activity. Inclusion of specific art therapy tasks designed to encourage expressions, discussion, and problem-solving in response to the emotional burden of chronic illness.	A variety of materials/techniques were offered, including clay, papier-mâché masks, paint, paper decoration forms, and markers.	Patients are encouraged by the art therapist to express their thoughts and feelings through art materials and interventions.	Helps to cope with troubling feelings and to master a difficult experience; experiences and feelings can be expressed and understood; being able to establish distance between themselves and their medical concerns; processing emotions through art, understanding that their problems are separate from themselves and that the children have an identity outside of their illness
Beh-Pajooh et al. (2018)	The subjects had a white sheet of paper to paint freely. At the end of each session, the students explained their painting briefly in the group.	Painting equipment: marker, color pencil, crayon, gouache, and water; white piece of paper	No information given	Effective because it is enjoyable for children; able to express their emotions (e.g., grief, fear and anxiety), feelings (e.g., whishes), and thoughts through projection, which leads them to achieve social adjustment
Chapman et al. (2001)	The CATTI (Chapman Art Therapy Treatment Intervention) begins with a graphic kinesthetic activity, followed by a series of carefully worded directives to elicit a series of drawings designed to complete a coherent narrative about the event (trauma). After completing the drawing and verbal narrative, the child is engaged in a retelling of the event using the drawings to illustrate the narrative.	Minimal art media	Children's emotional expressions are validated by the therapist as normal responses to the traumatic event to universalize their experience and reduce anxiety. During the retelling, numerous issues are addressed, including but not limited to misperceptions, rescue and revenge fantasies, blame, shame and guilt, coping strategies, treatment and follow-up plans, traumatic reminders, and reintegration strategies.	Facilitation of the integration of the experience into one's larger, autobiographical life narrative; facilitating the expression and exploration of traumatic imagery as it emerges from memory and finds form; utilizing the integrative capacity of the brain by accessing the traumatic sensations and memories in a manner that is consistent with the current understanding of the transmission of experience to language
Freilich and Shechtman (2010)	The child undergoing the therapy selects a topic, and the materials, for a project of interest. When necessary, conducting role-playing and guided discussions are used to increase efficacy.	Materials needed for art projects, such as paper, paints, pictures, journals.	The therapist assists and supports the youngster in carrying the project out. The therapist's role is to help the child identify a meaningful experience, a difficulty, or a conflict. In the process of working on the art piece, the therapist encourages the child to express related feelings and concerns, to explore them, and to reflect on them.	The subject(s) selected in art therapy is a reflection of important issues in the child's life that cannot be expressed directly; reflection leads to the development of insight the selection of goals for change; focusing on emotional exploration of difficulties; identifying problems; sharing problems with the therapist; cathartic experiences that lead to an increase in self-awareness and insight; focusing on an exploration of emotions and reflecting on them
Hashemian and Jarahi (2014)	Painting therapy (not further described)	Not described	Not described	Adjustment to their surroundings and therefore changing their inappropriate behavioral patterns; indirect communication with children
Kymissis et al. (1996)	Synallactic Collective Image Therapy (SCIT): drawing of a picture. Afterward, a brief presentation and voting one for discussion. The originator gives a title, offers association to it, and says how he/she felt before and after drawing. Other members give their associations. The resulting overlapping of the patient association was the collective image, which represented the basic theme of the session.	Drawing on 12 by 18 inches construction paper with pencils and colored markers.	The therapist takes an active role, directing and encouraging group members in the art activity. The therapists made sure group rules for orderly behavior were maintained.	The opportunity to freely present thoughts and feelings in a non-verbal way, within the structure of the group; the availability of the drawing as a non-verbal channel of communication helping regulation of the level of anxiety, enjoying the group; using artwork facilitated the group process
Liu (2017)	All sessions contain two domains: externalizing the problem and finding solutions. The experience associated with stress is drawn on small white paper. The future solution contents will be drawn on colorful, larger paper. All artworks were gathered and reviewed at the end of the intervention (last session) together with the parents.	Small white paper and large colorful paper; a variety of materials/techniques was offered, including drawing, painting, stress ball making, paper cutting, and paper folding	The therapist asks for what is better. The clients “stated needs for today” are related to overall goal(s) for therapy. The client is complimented for his strengths/resources. The therapist askes exception/difference questions. Scaling questions are being asked. Coping questions related to the client's abilities that emerged are being asked. Feedback on the helpfulness of the session is asked. The miracle question is being asked. The client is asked, “what else” was better in today's session. The families are given compliments about their contributions as the session ended. The client is asked to draw what they wish to draw/make but related to their problems. The therapist elicits the client to talk about the drawing and express their feeling. The therapist embeds most of the solution-focused questions and skills in the art-making process and guides the conversation. The therapist monitors that the drawings or the handcrafting are related to the intervention goal and that the session's drawing is focused on future, positive, and brightness.	SF-AT is a combination of Solution Focused Brief Therapy with Art Therapy. SF-AT group therapy in this study adopts a synthesized version of the constructivism theory and psychodynamic theory. This study also uses systems theory to frame the design of SF-AT and elucidate the mechanism of change. SF-AT addressed anger, stress, and emotional issues; non-judgmental acceptance and unconditional care to build rapport and facilitate change; therapeutic alliance; a practice promoting strength-based and positive perspective; empowerment by the strength-based treatment; positively construct information and experience; through positive cognitive construction and social construction, clients can build a new way of problem viewing and solving; this can help with negative thoughts and social impairments
Lyshak-Stelzer et al. (2007)	Trauma-focused art therapy (TF-ART). Scripted trauma-specific art activities (directives) for each session. Each participant completed at least 13 collages or drawings compiled in a handmade book format to express a narrative of his or her “life story.” The activities sought to support the youth in reflecting on several questions: What is the difference between feeling safe and unsafe with (a) your peers in the hospital; (b)peers on the street; (c) a staff member; (d) adults in your community; (e) peers at home; and (f) adults at home? When are feelings of fear and anger helpful, and how can they lead to increasing safety? What makes a place safe or dangerous? Can you contrast dangerous activities that you have engaged in during the past with safe activities? What made them safe or dangerous? A second phase of the protocol focused on sharing trauma-related experiences and describing coping responses. On the last session, each presented the book in its entirety to his or her peers.	Collage technique, drawing; making a book	Each adolescent was asked at the beginning of the session to do a “feelings check-in” describing how he or she was feeling in the moment using a single word or sentence, and a “feelings check-out” at the end of the session. After the art-making period, during which minimal discussion took place, the youth were encouraged to display their artwork to peers. They were encouraged but not required to discuss dreams, memories, and feelings related to their trauma history and symptoms. A second phase of the protocol focused on sharing trauma-related experiences and describing coping responses. For example, they were asked to share “some of the words that others have used to hurt you or help you in the past”; to describe nightmares, bad dreams, distressing memories, and flashbacks, as well as strategies used to cope with them; and to discuss traumatic “triggers” that served as reminders of trauma memories or feelings, along with coping strategies.	Exploring fundamental experiences associated with safety and threat; creating an opportunity for ways of orienting to safe and dangerous situations using non-verbal representations; imaginal representations used as the basis for verbalizing the associated experiences in a supportive social context; art products as a starting point for sharing traumatic experiences reduces threat inherent in sharing experiences of trauma by permitting a constructive use of displacement *via* the production of imagistic representations
Ramin et al. (2014)	A diversity of topics and means. Including: Image-making and imaginary drawing. Children started to image-making then draw whatever they prefer in their imaginary area; Children play-act and draw simple bad/good feelings in the group setting; Overall, drawing and discussing/exploring the result. At the ninth session, all the children worked on a group project to bring closure by drawing a ceremony on a large paper together with comments. At the end, a small exhibition of artwork was made.	Drawing on large paper, not further described.	An active role of the art therapist, for instance, recognizing dysfunctional ideas and beliefs children hold about themselves, their relations or interactions with the environment, and helping children identifying and restructuring them by using self-monitoring, problem-solving strategies, and learning coping responses and new skills.	A cognitive-behavioral approach. Non-verbal expression that is possible in art therapy is a safe way; imagination in combination with art-making; in art therapy, children can manage difficult emotions such as anger; art therapy can improve emotional understanding and anger management; in art therapy interventions children can learn coping responses, new skills or problem-solving techniques, increasing sense of belonging, to offer a non-threatening way and to communicate complex feelings and experiences.
Regev and Guttmann (2005)	The participants in control group B (*art therapy group*) created art projects, which were handled in an art-therapy fashion. Each meeting was divided into two parts. In the first 20 min, the children could freely choose to work with any of the available art-project materials and create (or continue to create) whatever they wanted. Then the work would stop, and the children would gather in a circle to discuss a child's (in turn) project.	A variety of materials	The art therapist supervised the group. Led by the art therapist, the discussion focused on questions such as: “how was the project done,” what it reminded the creator of, if it was similar to or different from other projects that he/she had made, if it reflected the way he/she felt that day, if it reflected anything that was happening in his/her life, and what he/she could learn from the project about himself/herself.	Artwork as a medium for self-understanding; artwork as a defense mechanism; artwork helps to ease personal difficulties; artwork helps to achieve emotional relief; artwork helps to achieve positive self-concept
Richard et al. (2015)	The intervention includes four sets of facial features (eyes, noses, mouths, and brows) representing four different emotions (happiness, sadness, anger, and fear), as well as a mannequin head. The participant was asked to create four different faces, representing happiness, sadness, anger, and fear. The participant was directed to choose a mouth, nose, eyes, and brows (in that order) that represented the correct emotion.	Four sets of facial features (eyes, noses, mouths, and brows); a mannequin head. Facial features were molded with Super Sculpey. Scotch, Adhesive Putty was used to attach the facial features to the Styro Full Blank Head. On the head paint was applied.	The participant was directed to choose a mouth, nose, eyes, and brows (in that order) that represented the correct emotion. For example, the researcher asked: which one of these mouths do you think would be a happy mouth? The participant received two attempts at choosing the correct feature. If the correct feature was chosen, the researcher responded: yes, that is a happy mouth! If an incorrect feature was selected, the participant was redirected with a statement such as: I do not think that is a happy mouth. A happy mouth has ends that turn upward. Then the participant made a second attempt at selecting the correct feature. If this attempt also failed, the therapist directed the participant to the correct feature.	By using three-dimensional materials to recreate emotions with facial features first the Kinesthetic /Sensory level is engaged through touch, next the Perceptual/Affective level is activated as the face is directly constructed with the materials, and possibly the Cognitive/Symbolic level can be mobilized to reinforce the identification of emotions; art activities involving tactile experiences help dissociative children connect through the ability to touch and create; information processing occurs at each of the first three levels on the ETC: Kinesthetic/Sensory, Perceptual/Affective, and Cognitive/Symbolic
Rosal (1993)	Two forms of art therapy: *Art as therapy group (a) (experimental)*: unstructured, the children were encouraged to use the media and be creative with them. *Cognitive-behavioral art therapy (b) (Control)*: use of specific objectives, a stated theme, specific media, and discussion topics. Basic structure: muscle relaxation, imaginary activity, clean up, discussion. Use of cognitive-behavioral principles: behavior contingencies, imagery, modified desensitization, problem-solving techniques, relaxation, stress inoculation, verbal self-instruction.	In both groups, the materials ranged from paint, drawing pencils and pens to clay, collage and construction parts.	*Art as therapy group (a)*: The therapist was active, yet nondirective, by controlling the environment through manipulations of ambiguity and anxiety. Therefore, if tensions in the group were brought to a dangerous level, the therapist intervened through clarifying issues and helping the group find alternatives to the problem. The therapist also assisted any child who was having difficulty with a specific medium. *Cognitive-behavioral art therapy group (b)*: delineated verbal instructions, directions for art media.	The intervention *art as therapy* could change LOC perceptions through the process of creating art and the experience with the art as a vehicle for discussion and feedback from others. The type of group therapist behavior was based on Whitaker and Lieberman's interpersonal interaction approach to group therapy. Intervention concerning cognitive-behavioral art therapy. The act of producing art may reinforce or enhance internal LOC (Locus of Control) perceptions; each line placed on a paper is the direct result of the child's behavior; A child's movement is reinforced visually by the mark that is produced; there is a direct link between behavior and outcome; drawings are derived from inner experiences The inner experiences may be perceptual, emotional or cognitive processes that are transformed into visual display Without even examining the content, a drawing is a tangible record of internally controlled behaviors; in art therapy, these tangible records are discussed, further reinforcing a child's inner experience.
Schreier et al. (2005)	Chapman Art Therapy Treatment Intervention (CATTI): one-to-one session at the child's bedside, completion 1 h. Starting: drawing activity. Drawings are used, creating a narrative about the event; the child can discuss each drawing. Then a retelling of the event, using the drawings to illustrate the narrative.	Minimal art media for drawing.	The child is encouraged to discuss each drawing. During the retelling, numerous issues are addressed, including misperceptions, rescue and revenge fantasies, blame, shame and guilt, coping strategies, treatment and follow-up plans, traumatic reminders, and reintegration strategies.	The art intervention offers an opportunity for the child to sequentially relate and comprehend the traumatic event, transport to the hospital, emergency care, hospitalization, and treatment regimens, and post-hospitalization care and adjustment; the drawing activity is designed to stimulate the formation of images by activating the cerebellum.
Siegel et al. (2016)	Patients selected buttons, threads, and words with which they constructed their Healing Sock Creature. The imaginary creatures were sewn and stuffed with magic bean, sand, or fiberfill. Children placed wishes inside the Healing Sock Creature to express their feelings.	Unused hospital socks and small kidney dishes to place buttons and threads. Sewing materials, magic beans, sand, fiberfill.	The therapist becomes the co-creator under the direction of the child by forming a bond of trust as the child shares their design ideas, which may include conversations about symbolic meanings of buttons or colors or threads.	The creative process, exploring deeper meanings in a patients experiences; integrating psychotherapy with multi-arts, the intermodal approach can help children access, process, and integrate traumatic feelings in a manner that allows for appropriate resolution, to reduce stress; this therapy uses imagination, rituals, and the creative process; a symbol can hold a paradox that the rational mind cannot fully explain; choosing a special button or writing a wish mirror, characterizes the child's psyche at this crucial moment; it enables the child to visualize and let go of troubling and unanswerable questions, thus relieving suffering
Tibbetts and Stone (1990)	Individual artwork (not further described).	-	The central focus of the art therapist was to assist the subjects to increase in the present their sense of personal power and responsibility by becoming aware of how they blocked their feelings and experiences anger. The approach was non-interpretive, with the participants creating their direct statements and finding their meanings in the individual artwork they created.	Non-interpretive. The art therapy approach utilized in the present study was consistent with the principles of gestalt. The primary role of the therapist as listening, accepting, and validating; art therapy is an integrative approach utilizing cognitive, motor, and sensory experiences on both a conscious and preconscious level; it initially appears less threatening to the client.
Jang and Choi (2012)	Each session had the following phases: introduction, activity, and closing. In the introduction phase, the participants greeted one another, did some warm-up clay activities, and were introduced to theme-related clay techniques. In the activity phase, they did individual or group-based activities making shapes using clay. In the closing phase, feedback about their performance was exchanged.	Clay	The art therapist asks questions. No further information provided.	To shed a sense of helplessness or depression with their physical movement of patting or throwing clay pieces in the activities; the continued and repeated experience of pottery-making throughout the sessions contributed to bringing about a positive change in the regulation and expression of emotions; the plasticity of clay made it easy for the participants to finish their clay work successfully; curiosity, toward the process through which a clay piece was transformed into glassy pottery and molding techniques or kiln firing that were learned in each session were factors that contributed to the positive changes; the plasticity of clay also enabled the participants to get a sense of control over the material because they could change the shape as they wished, which contributed to a positive evaluation of their own performance; witnessing the transformation of a piece of clay to complete, glassy pottery, combined with the positive feedback given to the participants, caused a sense of achievement and optimistic outlook on the future.
Khadar et al. (2013)	Painting therapy	Not described	The art therapist is present and does not impose interpretations on the images made by the individual or group but works with the individual to discover what the artwork means to the client.	The child makes art in the presence of his or her peers and the therapist, this exposes each child to the images made by other group members on both a conscious and an unconscious level; to learn from their peers and to become aware that other children may be feeling just like them; make meaning of events, emotions or experiences in her life, in the presence of a therapist; the process of drawing, painting, or constructing is a complex one in which children bring together diverse elements of their experience to make a new and meaningful whole; through the group, they learn to interact and share, to broaden their range of problem solving strategies, to tolerate difference, to become aware of similarities and to look at memories and feelings that may have been previously unavailable to them; the image, picture or enactment in the art therapy session may take many forms (imagination, dreams, thoughts, beliefs, memories, feelings); the images hold multiple meanings and may be interpreted in many different ways.
Khodabakhshi Koolaee et al. (2016)	First session: Initial introduction, declare short objective of sessions. Second session: Collaborative painting among therapist and child: make a closer contact with children. Third session: Technique of children's scribble: reduce resistance and anxiety in children. Fourth session: Photo collage: Increase cooperation during the treatment process. Fifth session: Drawing with free issue: emotional discharge. Sixth session: Drawing the atmosphere of the hospital and the inpatient portion: express anxiety of children related to atmosphere hospital. Seventh session: Drawing family as animal: evaluate the attitude and relationship of children with family. Eighth session: Anger collage for expressing children's anger and aggressiveness. Ninth session: Drawing with free issue: express emotion. Tenth session: Evaluate the effectiveness of drawing on aggressiveness and anxiety. Eleventh session: Follow-up session.	Photo collage, drawing. Not further described.	Not described	Painting provides opportunities to communication and non-verbal expression; it can serve as a tool to express the emotions, thoughts, feelings, and conflicts; anxiety symptoms of children emerge in metaphorical symbols such as play and painting; drawing permits the children to convey their thoughts and dissatisfaction with environment-related to the hospital, they can express their emotion in safe atmosphere: drawing improves anger management and emotional perception with learning the accurate coping response, the techniques and problem-solving skills, and provides the non-invasive way to communicate in a complex emotional situation.
Pretorius and Pfeifer (2010)	Four themes: (1) Establishing group cohesion, and fostering trust by group painting, guided fantasy with clay, and story-making through a doll. (2) Exploration of feelings associated with the abuse by drawing feelings, drawing perpetrators, placing of these in boxes. (3) Sexual behavior and prevention of revictimization by role-playing and mutual storytelling (4) Group separation by painting, drawing, or sculpting feelings associated with leaving the group	Paint and drawing materials, not further specified, and clay.	Therapeutic behavior based on the existential-humanistic perspective, and incorporated principles from Gestalt therapy, the Client-centered approach, and the Abuse-focused approach	Group psychotherapy can ameliorate difficulties encountered in the use of individual therapies with sexually abused children, including an inherent distrust of adults, fear of intimacy with and disclosure to adults, secrecy and defensive behavior; group therapy also offers children the opportunity to realize that they are not alone in their experiences and that other children have had similar experiences, this realization may be a great source of relief that helps reduce the sense of isolation; art therapy involves a holistic approach in that it not only addresses emotional and cognitive issues but also enhances social, physical and developmental growth; art therapy appears to help with the immediate discharge of tension and simultaneously minimize anxiety levels; the act of external expression provides a means for dealing with difficult and negative life experiences; art therapy, therefore, not only assists with tension reduction but also with working through issues, thereby leading to greater understanding; Group art therapy acknowledges the concrete thinking style of latency-aged children and accordingly provides an opportunity for non-verbal communication; contact with group members may also decrease sexual and abusive behaviors toward others.
Ramirez (2013)	Six interventions were repeated twice: (1) Predesigned mandala template/complete design. (2) Create self-portraits (3) Design a collage. (4). Mold clay into a pleasing form, which could be an animal, a person, an object, or an abstract form. (5) Visualize a landscape from imagination and paint it. (6) Arrange a variety of objects in a pleasing orientation and draft the still life with a pencil	Color pencils, markers, crayons, and oil pastels; charcoal, ink, or mixed media, clay; acrylic paints or watercolors	The therapist facilitates the creation of the artistic product and is supportive. The art therapist suggests expressive tasks in a manner that shows respect for their way of reinventing meaning and involves subject matter that is of interest to the teen.	The creative process involved in artistic self-expression helps people to become more physically, mentally, and emotionally healthy and functional, resolve conflicts and problems, develop interpersonal skills, manage behavior, reduce stress, handle life adjustments, and achieve insight.
Steiert (2015)	The given theme was Heroes; no further information provided	An extensive range of different materials was available. There was wood, stone, plaster, and a comprehensive selection of paint and drawing materials, also felt and other textiles.	Decisions on what to do, which materials to use were discussed with the patients. The shaping of the heroes was supported by talking about this, viewing comics, searching images, watching videos. For dealing more intensely with the heroes, suggestions from the therapist were given.	The processing on a symbolic, playful and imperious level, gives the child the opportunity for a gradual approach for their conflicts, without defense mechanisms undermining it; heroes and heroic stories support children and adolescents not just in their childhood development but have potentially also a positive influence on processing disease; in the children and adolescents can find their emotional reality and find solution options for the handling of these conflicts' themes.
Wallace et al. (2014)	Expression of feelings by mandala drawing and painting. Exploration of changes in family functioning by a family drawing with pencils, markers, and oil pastels.	Paint, not further specified; drawing materials such as pencils, markers, and oil pastels	The art therapist provided general art therapy guidelines such as that there are no mistakes or a right or wrong way to express themselves in art. The art therapist encouraged the participants to explore the art materials, to relax, and to have fun. The art therapist inquired about colors and feelings depicted and asked the participant to share examples of why each feeling had been or was being experienced. The art therapist normalized and validated the siblings' feelings, provided support and empathy.	Art therapy may offer a non-verbal means of communication, an emotional outlet, and a source of empowerment and control for this population; art therapy can assist children in communicating difficult feelings and in reducing symptoms of anxiety and posttraumatic stress; art therapy can stimulate the verbalization of hospital experiences and resolving anxiety and fear-provoking thoughts; art therapy offers an opportunity for making choices; the process of creating art may provide a sense of control for siblings during a time when many decisions are beyond their control; the intervention group had significantly lower PTSS, it appears that the siblings gained mastery and processed their emotional responses; art therapy assisted in reintroducing control into the healthy siblings' life and allowed them to express and process the challenges and changes that they were experiencing; art therapy allowed the siblings to express emotion without resorting to words; art therapy seems to have assisted the healthy siblings in gaining a level of comfort that facilitates asking questions, which can result in an effective educational intervention.
Walsh (1993)	AFI (art future-image intervention): Clients met together, formulated plans for a future identity, and created a future self-image caricature poster from an enlarged polaroid photograph and a career/body-image packet designed by the researcher.	Polaroid photos; drawing materials.	Not described	Identity formation; promotion of qualities associated with psychological health: (a) exploration of various career options, (b) decision-making, and (c) identity achievement.
Chaves (2011)	Creating therapeutic art books	A diversity of materials, not further specified	The art therapist offers the individual social support; the therapist never criticized the participants work, attempted to avoid giving art instruction, and created a safe and accepting environment.	The creation of art books is a way for individuals to “visually” document their journey throughout their hospitalization and recovery process. Through art-making, especially within the contained boundaries of a book, individuals are provided with a bridge that can help them move toward their authentic selves. Through books, individuals can track their recovery process chronologically, can review their emotional progress, and can choose what they want to create, without their creations being judged by others. The books are a definite shape, and the books can be closed, they act as a container for the individual's emotions, thoughts, and sense of self. The book becomes an investment in everyone's recovery and a reflection of their process.
D'Amico and Lalonde (2017)	The sessions employed art-based interventions using the art-based interventions focused on developing self-expression, creativity, and the consolidation of social skills through art-making, discussion, play, and collaborative projects.	Various two- and three-dimensional art materials	Therapists employed various art-based techniques and training strategies to increase student practice and performance of desired social behaviors. If any child required additional instruction for a particular social skill or problem behavior, a therapist addressed this in a more individualized manner in the therapeutic group session. The art therapists created a variety of opportunities for the children to cooperate and to build cohesion among group members. The art therapists used these and other therapeutic activities to help the children improve their social functioning and work through personal issues, while providing them with opportunities for behavioral practice to enhance self-esteem and well-being.	It provides opportunities to solve social problems visually and in a concrete and creative way; it offers a way to learn information in an unconventional, non-verbal, comprehensive, and expressive manner through rich sensory experiences with a variety of art materials; the combination of art and therapy is pertinent to address the individual's feelings of anxiety, depression, and frustration through empathetic listening, visual feedback, and using creative projects to build a trusting relationship; art therapy can empower children to become active participants in their treatment, and to use their creativity in a meaningful and productive manner.
Devidas and Mendonca (2017)	Art therapy included theme drawing, theme painting, making future portrait, freehand drawing, clay modeling, scribble drawing, paper bags making, preparing stuffed toys, finger painting, and attach a drawing to a balloon.	Drawing and painting materials including finger paint (not further described); clay, materials for making paper bags; stuffed toys	Not described	Art can raise the self-esteem and promote psychological comfort; Art is the language of mind; emotions and feelings can be best expressed through art
Epp (2008)	Conversation skills are practiced in an unstructured manner (10 min), with leading questions. Then a structured art activity (30 min), with instructions and next unstructured free time (20 min).	A variety of materials and activities is used.	Cognitive-behavioral strategies are used throughout the group therapy session. An example of this would be a therapist asking a student, “When you are frustrated/happy, what do you say to yourself? What is your self-talk?” Usually, the group is led in a brainstorming exercise to discover ways to change self-talk to improve feelings or make better choices \with difficult feelings. Social skills are “taught” by therapists who carefully watch how children approach or do not approach each other, intervening in a helpful, non-threatening, concrete manner so that the children learn how to structure their playtime in a social context.	Cognitive-behavioral strategies are used. Through the child's art, the therapist can gain insight into what the child is experiencing, which is information that is not readily available through verbal means; art therapy as a component to social skills training may increase the willingness of children to participate because art is an activity that they find acceptable; art therapy offers a way to solve problems visually; it forces children with autism to be less literal and concrete in self-expression; it offers a non-threatening way to deal with rejection; it replaces the need for tantrums or acting-out behaviors because it offers a more acceptable means of discharging aggression and enables the child to self-soothe; use of icons, symbols, and social stories help the children to remember what they were taught; when children and therapists collaborate to custom-make these symbols, icons, and stories for each child's unique challenges and goals, the children take ownership of them and integrate them into their internal experience; Comic strips are drawn by the teacher and then used to “teach” to the children, with discussion and analysis of the portrayed events. Children who are visual learners take in this information in a way that stays with them
Hartz and Thick (2005)	The specific art therapy interventions used during the study included magazine collage and yarn basket-making. The same projects and an identical selection of materials were provided to all participants, regardless of the intervention approach. All participants received group therapy with their core group 5 days a week as their primary treatment and participated in several adjunctive therapies weekly (including art therapy). A majority also had monthly family therapy sessions, facilitated by their core-group leader.	A variety of materials, including magazines and yarn, for basket making.	In the *art as therapy (a)* approach, the design potentials, technique, and the creative problem-solving process were highlighted. Artistic experimentation and accomplishment were emphasized. In the *art psychotherapy approach (b)*, a brief psychoeducational presentation was employed, and abstraction, symbolization, and verbalization were encouraged. During facilitation, personal awareness and insight were emphasized.	*Art as therapy* (a) focuses on developing mastery, creating structure, and sublimating conflicts to strengthen the ego; the confidence and authenticity that participants reported suggest the development of meaningful and supportive relationships; experiencing growth and mastery in art therapy provided participants with an experience of success and pride transferable to other areas of their lives. *Art psychotherapy* (b) is a cognitively based approach that emphasizes insight and involves some verbal processing of the art products
Higenbottam (2004)	Group activities included several spontaneous art sessions as well as some group directives inspired by the writings of Ross (1996); Ruiz (1997); Riley (1999), as well as Daley and Lecroy's (2001).	Not described	The art therapist is a group facilitator and a cultural mediator. Encouraging the client's creative expression as well as teaching and modeling coping skills. The students were given opportunities to make their own decisions during the group, and there were no group rules *per se*. The therapist set some rules to prevent art therapy time being used for gossip. Handouts with developmentally appropriate or topical information were given. As the group evolved and certain subjects or questions arose, handouts evolved to reflect this.	Art therapy is a modality that commonly diminishes adolescent resistance; art therapy group for adolescent females, focusing on self-esteem and body image can provide the opportunity for action, expression and discussion at a time when such explorations are most important to development; adolescent art therapy groups provide a necessary forum where teenagers can safely express themselves, exert independence and make safe decisions.
Jo et al. (2018)	A manualized art intervention program: Sessions 1 to 3 consisted of the “getting to know” stage, sessions 4 to 8 were the “releasing” stage, and sessions 9 to 12 were the “soaring” stage. The “getting to know” stage focused on easing the tense atmosphere by building a rapport between the therapist and children, as well as helping the children become more familiar with the material and gain an awareness of their inner self. The “releasing” stage was designed to help the children experience the freedom of feeling and relieving their pent-up feelings through expression. It also dealt with their relationships with the parents and siblings within the family. The “soaring” stage focused on providing positive feedback in the form of hope for the future and positive awareness of one's current self.	All three stages used a variety of materials and activities.	Not described	The art intervention is a tool that can be effective in helping people overcome difficult experiences and psychologically cope by encouraging emotional expression *via* art; this intervention can be particularly effective in children since it allows those who are unable to accurately verbalize their thoughts and feelings to convey these more comfortably; it is assumed that a significant improvement in self-esteem might help children recognize themselves positively through expressing their feelings freely and offer greater emotional support; the externalizing problems scale and its aggressiveness subscale, emotional instability, and other categories showed a significant decrease, which is believed to be the result of the children sublimating their aggressive energy through their artwork, resulting in greater emotional stability.
Pifalo (2002)	Sessions 1 and 2: Group puzzle mandala. 3: Creation of two lists of feelings. 4: Creation of a “container” for these feelings/several materials. 5: Drawing of “roadmaps.” 6: Puppet making. 7: Clay representation of significant people. 8: Making of a bracelet. 9: Designing a safe place. 10. Sharing creations of products in the group	A variety of materials, including puzzle mandala. Drawing materials. Materials for making a puppet; clay; materials for making a bracelet	The art therapist “offers a container” that allows the freeing up of visual or kinesthetic imagery, and still allows sufficient emotional distance from overwhelming pain. The art therapist acts as a witness to the trauma. The role of the art therapist was to facilitate the negotiation of a safe passage between the poles of constriction and intrusion in discussions between the girls.	A combination of art therapy and group process. The images that participants created individually and as a group gave a voice to the powerful emotions that they had previously suppressed; the stultifying bonds of silence and secrecy- the powerful weapons of the perpetrator-were broken as each girl found the courage to identify her feelings and speak them aloud within the safety of the group; both the group members and the images that they created bore witness to their rage, grief, pain, and loss; the artwork allowed the group members to create containers for their rage and their tears.
Pifalo (2006)	A combination of art therapy, cognitive behavioral therapy, and group process to address the therapeutic issues related to childhood sexual abuse. Not further specified.	A variety of materials, not further specified.	Not described	Cognitive-behavioral therapy offers clear-cut goals for trauma-focused therapy; art therapy “cuts to the chase” in a way that talk therapy alone cannot because art therapy does not rely strictly on a verbal mode of communication; art therapy is uniquely suited to promote basic goals of crisis intervention involving cognition and problem-solving, and ventilation of affect; the use of image-based interventions such as creating containers to express and release powerful emotions, making maps to organize a coherent trauma narrative and set future goals, using multiple media to illustrate the photographic nature of traumatic memories, and graphically representing internal and external sources of support, provides an opportunity for traumatized children to express what they may not yet be able to verbalize.
Rowe et al. (2016)	The art therapy interventions delivered within the sessions were tailored to the client's specific needs and therapeutic goals, as established by the client, the family, and the therapist (not further described).	Not described	Art therapists worked with their clients to form therapeutic goals during initial sessions, followed by both structured and unstructured weekly art therapy sessions (not further described).	Art therapy is an effective psychotherapy for traumatized individuals based on the theory that trauma is stored in the memory as imagery, and art-making is an effective tool for processing these images; ATI's school-based art therapy program is uniquely suited to serve its adolescent refugee clients because a perceived sense of safety at school and of school belonging protects against PTSD, depression, and anxiety; the program seeks to develop clients' strengths as well as ameliorate negative symptoms associated with the refugee experience, such as depression and anxiety
Saunders and Saunders (2000)	Not described. Different for all individual subjects.	Not described	The therapeutic relationship between client and therapist is one that must be developed and nurtured by the art therapist to facilitate the therapy process. Both the product and the associative references may be used by the therapist to help the client find a more compatible relationship between his/her inner and outer world. A positive therapeutic relationship. Ultimately, the art therapist guides his/her client through to the therapeutic goals determined by the nature of the client's assessed needs.	Art therapy uses the modality of art media to help clients express their thoughts, feelings and experiences; the use of art as therapy implies that the creative process can be a means of both reconciling emotional conflicts and of fostering self-awareness and personal growth; creating a work of art provides the client with a vehicle for self-expression, communication, and growth; process, form, content and/or associations become important for what each reflects about personality development, personality traits, and the conscious behavior and unconscious motivation; art therapy is the modality of choice for helping children, and adults, who find it difficult to verbalize their feelings and to acknowledge them to themselves because of their age, developmental level, lack of trust, fear of acknowledging the unknown, or mental illness. One of the central features of art therapy is its ability to help children become more communicative about their feelings and less likely to either internalize them in unhealthy ways or to act them out in destructive ways.
Sitzer and Stockwell (2015)	Students engaged in art therapy combined with CBT and DBT modalities. Students learned to listen, describe their artwork and personal experiences, give feedback and encouragement to their peers.	A variety of materials and activities was used; not further described	The art therapist provides a safe and protected space so that the child can model the experience of positive affect regulation.	Art therapy combined with CBT and DBT modalities. Communication skills are the main vehicle of change, from the development of trust in module one, through mindfulness module six; students learned to listen, describe their artwork and personal experiences, give feedback and encouragement to their peers; art therapy provides the medium and expressive capacity to elicit several positive resilience characteristics; the activation of positive emotions, increasing emotional self-efficacy, and improved self-esteem walks lock-step with resilience; Mindful mandalas are a quiet, non-verbal art directive designed to facilitate a meditative experience; post-artwork discussion includes review all skill-sets taught in the program: identification of beliefs that contribute to optimism, emotional regulation skills, and stress management, communication skills, mindfulness thinking.
Stafstrom et al. (2012)	Each session included a different discussion topic and art activity designed to enhance positive adjustment to epilepsy. (1): Self-portrait inside/outside box, drawing, and collage. (2): A memory or feeling about epilepsy, drawing and painting media. (3): Mandala of personal symbols, drawing media (4): A dream or goal for the future, diorama with digital photo portraits, mixed media.	Drawing materials; materials to make a collage; painting materials; digital photos; mixed media	Each session is facilitated by an experienced art therapist (not further described).	Art is a projective technique that can be used to assess the emotional and psychological challenges that affect children and adolescents; artwork allows children to express their feelings in a way that they may find difficult verbally; artistic creations contain symbols and metaphors that articulate the importance of a disorder in a child's life and experience; children often find expression through art to be empowering and enjoyable.

*ATI, Art Therapy Institute; CBT, Cognitive Behavioral Therapy; DBT, Dialectic Behavioral Therapy; ETC, Expressive Therapies Continuum; LOC, Locus of Control; PTSD, Post Traumatic Stress Disorder; SF-AT, Solution Focused Art Therapy*.

#### Materials/Techniques

Regarding the category art materials/techniques, three subcategories were found. In the first subcategory, only two-dimensional art media/techniques were used, such as drawing, painting, or printing (the art product possessed length and width, but not depth). Used as materials were for instance, (acrylic) paint, markers, color pencil, crayons, gouache and water, white pieces of paper, cardboard, construction paper with pencils and colored markers, a “sketch” coloring, pencils, markers, and oil pastels (1, 3, 6, 7, 10, 14, 18, 21, 23). No specific art techniques concerning the way the materials were applied were mentioned in this subcategory. In the second subcategory, both two-dimensional and three-dimensional art media and techniques (art that can be defined in three dimensions: height, width, and depth) were offered: clay, papier-mâché masks, paint, paper decoration forms and markers, pictures and journals, paper, cardboard, construction materials, hospital socks, buttons and threads, sewing materials, magic beans, sand, fiberfill, photos, wood, stone, plaster, felt and other textiles, and yarn. In this subcategory, specific art techniques were mentioned, such as paper cutting and paper folding, collage technique, bookmaking, building a face, basket-making, clay techniques, guided fantasy, group painting, story-making through a doll, placing feelings in boxes, drawing/sculpting feelings, making clay shapes, creating self-portraits, and molding clay (2, 5, 8, 11, 13a, 19, 20, 22, 26, 27, 28, 29a, 29b, 30, 31, 32, 33, 36, 37). In the third category, both two-dimensional and three-dimensional art materials/techniques were applied, which matched the specific assignment or topic given (4, 9, 12, 13b, 14, 15, 17, 24, 25). For instance, drawings were made, and the collage technique was used to make a book (9). Four sets of facial features (eyes, noses, mouths, and brows), as well as a mannequin head, were offered for representing facial emotions (12), and in one study, patients used buttons, threads, and sewing materials with which they constructed their Healing Sock Creature, which the children filled with magic beans, sand, or fiberfill (15).

#### Topics/Assignments

Three subcategories were found concerning the category topics/assignments. The first subcategory, *free working with the materials without topics/assignments given*, was applied in five AT interventions (3, 5, 11, 13a, 16). In the second subcategory, 26 AT interventions *used assignment(s) or gave topics* (1, 2, 4, 7, 8, 9, 10, 12, 13b, 14, 15, 17, 19, 20, 21, 22, 23, 24, 25, 27, 29 a/b, 32, 33, 36, 37). The third subcategory concerned combinations of these two. Two studies mixed free working and giving topics/assignments (28, 30), and seven studies did not describe the intervention explicit enough to classify them (6, 18, 26, 31, 34, 35, 36). A wide range of activities based on topics and/or assignments were reported. Eleven categories could be detected; (1) *getting familiar with the art material* (1, 17) like “learning about art media” (1) and “warm-up clay activities and introduction to theme-related clay techniques” (17); (2) focusing on *family perspective*, like for instance, “draw first childhood memory/family relations”(1, 23), drawing family as animal (19); (3) *working with visualization, fantasy, and meditation* (1, 10, 20, 21), such as guided fantasy with clay, and story-making through a doll (20); (4) *expressing emotions* (1, 14, 19, 20, 23, 32) like “the participant was asked to create four different faces, representing happiness, sadness, anger, and fear” (14) or “make an anger collage” (19); (5) *focusing on specific problems such as chronic disease or stress-related events* (2, 4, 8, 9, 14, 15, 19, 37) such as “the experience associated with stress is drawn on small white paper and the future solution contents will be drawn on colorful, larger paper” (8) and “drawing feelings, drawing perpetrators, placing of these in boxes” (19); (6) applying *group activities* (10, 19, 20, 32), for instance, “make a group painting”(20) and “all the children were asked to work on a group project to bring closure by drawing a ceremony on a large paper together with comments” (10); (7) *working on an exhibition of artwork* (10, 32), for instance, “at the end, a small exhibition of artwork was made” (10); (8) *focusing on the material/technique* (17, 21, 27, 37) such as “making shapes using clay” (17) and “mold clay into a pleasing form, which could be an animal, a person, an object, or an abstract form” (21); (9) *focusing on specific art techniques* (19, 21, 29) such as “arrange a variety of objects in a pleasing orientation and draft the still life with a pencil” (21) or “make a photo collage”(19); (10) *working with a product/object as a result* (24, 25, 27, 32) such as, for instance, “making a bracelet” (32), “making paper bags” (27), or creating therapeutic art books (25); (11) applying *general activities* (1, 7, 19, 22, 27, 32) like drawing of a picture (7) and “the given theme was heroes”(22). Two studies (13b, 33) gave assignments/topics but did not specify these.

#### The Role of Language

Three subcategories were found concerning the *role of language as a form of verbal expression* accompanying the use of art materials and techniques: *the produced artwork was mainly discussed afterward in a group meeting or on an individual basis* (1, 2, 3, 4, 9, 10, 11, 13b, 14, 17, 29, 36) or *feelings and concerns were mainly discussed and reflected on while working* (5, 12, 13a, 15, 18, 22, 25, 26, 28) and *other varieties* such as: the work was (verbally) presented (4) and/or patients also retold the narrative created (5). In one study (7), the originator gave a title, offered associations to it, and said how he/she felt before and after drawing other members gave their associations. In one study (8), all artworks were gathered as a collection and reviewed at the end of the intervention (last session) together with the parents.

### Therapist Behavior

Regarding therapist behavior, the information is structured in two categories: the *therapist behavior, including social interactions with the client(s)*, and *the handling of materials by the therapist, including material interactions with the client(s)*.

#### Therapist Behavior, Including Social Interactions With Their Client(s)

The information revealed *three* broad behaviors: *non-directive* behavior, *directive* behavior, and behavior that can be considered *eclectic*. Non-directive behavior refers to AT interventions in which the therapists showed mainly a following and facilitating attitude toward the children/adolescents. Thirteen AT interventions applied this kind of therapist behavior (13a, 15, 16, 17, 18, 20, 21, 22, 23, 25, 29a, 30, 36). Interactions with clients were for example, “the therapist was non-interpretive, with the participants creating their direct statements and finding their meanings in the individual artwork they created” (21) and “the therapist facilitates the creation of the artistic product and is supportive” (13a). Directive behavior refers to AT interventions in which the therapist showed an active and leading role toward the children/adolescents. Ten AT interventions (4, 8, 10, 11, 12, 13b, 24, 26, 28, 29b) used this kind of therapist behavior. Interactions with the clients were, for example, “the therapist asks exception/difference questions” (8) or “the participant was directed to choose a mouth, nose, eyes, and brows that represented the correct emotion” (12). A mix of these two types of therapist behaviors (eclectic) was applied in nine AT interventions (2, 5, 7, 9, 14, 32, 33, 34, 35), for instance: “each adolescent was asked at the beginning of the session to do a ‘feelings check-in’ describing how he or she was feeling in the moment and a ‘feelings check-out’ at the end of the session. In the art-making period, a minimal discussion took place” (9) or “art therapists worked with their clients to form therapeutic goals during initial sessions, followed by both structured and unstructured weekly AT sessions” (34). In seven AT interventions (1, 3, 6, 19, 27, 31, 37), insufficient information was given to classify the therapist's behavior.

#### The Handling of Materials by the Therapist, Including Material Interactions With the Client(s)

Information was provided by seven studies: “the therapist assists and supports the youngster in carrying out the activity” (5), “the therapist embeds solution-focused questions and skills in the art-making process” (8), “during working with materials, there was minimal discussion” (9), “the child was directed to choose features/materials that represented the correct emotion” (12), “the therapist gave delineated verbal instructions and directions for art media” (13a), “the therapist-assisted the child having difficulty with a specific medium” (13b), “the therapist became the co-creator” (15), and “the therapist avoided giving art instructions” (25).

### Supposed Mechanisms of Change

In the introduction and discussion sections of the articles, a range of supposed mechanisms of change as substantiation of the intervention and outcomes were described (Table 2). The supposed mechanisms of change could be categorized into two categories: *art therapy specific* and *general psychotherapeutic mechanisms of change*.

#### Specific Mechanisms of Change

Eight subcategories of a specific mechanism of change were detected. The first category was *Art therapy as a form of expression to reveal what is inside*. This large subcategory, could be divided into three forms: *art as a form of visualizing and communication in general* (1, 13, 15, 19, 20, 26, 28, 33, 35, 36), such as, “it enables the child to visualize” (15); *art as a manageable expression and/or regulation of emotions* (1, 2, 3, 7, 8, 10, 19, 20, 23, 27, 28, 31, 33, 35, 37), e.g., “through art emotions can be processed” (2); and *art as a way of expression through specific processes* (1, 4, 5, 9, 10, 11, 14, 15, 17, 19, 22, 25, 28, 29, 31, 32), for instance, “reduces threat inherent in sharing experiences of trauma by permitting a constructive use of displacement *via* the production of imagistic representations” (9). The second category was *Art therapy as a way of becoming aware of oneself*, mentioned by 10 studies (1, 2, 11, 13, 16, 23, 24, 25, 35, 36), for instance, “to regain a sense of personal agency” (1). The third category was defined as *art therapy as a way to form a narrative of life*, like “facilitation of the integration of the experience into one's larger, autobiographical life narrative” (4), while the fourth category dealt with *art therapy as integrative activation of the brain through experience*, which was mentioned in six studies (4, 12, 14, 16, 34, 26), for instance, “utilizing the integrative capacity of the brain by accessing the traumatic sensations and memories in a manner that is consistent with the current understanding of the transmission of experience to language”(4). The fifth category *art therapy as a form of exploration and/or reflection* was mentioned in seven studies (1, 9, 15, 18, 30, 5, 8), for instance, “to explore existential concerns” (1), and the sixth category *the specifics of the art materials/techniques offered in art therapy* was mentioned in three studies (13, 17, 30), for instance, “because they could change the shape as they wished, which contributed to a positive evaluation of their own performance”(17). The seventh category *art therapy as a form to practice and/or learn skills* was mentioned in four studies (10, 19, 28, 33), for example, “in art therapy interventions, children can learn coping responses, new skills, or problem-solving techniques” (10). Finally, the eighth category *art therapy, as an easily accessible, positive and safe intervention by the use of art materials* was mentioned by 15 studies (1, 2, 6, 8, 10, 16, 19, 23, 24, 25, 28, 29, 30, 32, 37), for instance, “non-verbal expression that is possible in art therapy is a safe way”(10).

#### General Mechanisms of Change

Two subcategories of general mechanisms of change could be defined. The first subcategory was defined as *art therapy as a form of group process*, mentioned by eight studies (7, 9, 13, 18, 20, 29, 30, 36), for instance, “present thoughts and feelings in a non-verbal way within the structure of the group”(7). The second, *the therapeutic alliance in art therapy*, was mentioned by six studies (5, 8, 16, 18, 26, 29), for instance, “the primary role of the therapist as listening, accepting, and validating” (16).

## Synthesized Findings

### Means and Forms of Expression and Therapist Behavior

Concerning the search for similarities and differences, the three found forms of therapist behavior were used to distribute the means and forms, which gave the following results.

#### The Therapist Behavior Was Non-directive

The therapist showed mainly a following and facilitating attitude toward the children/adolescents; in this category (*n* = 13), the use of means and forms of expression was variable, but most often, children and adolescents worked on base of topics and assignments with both two- and three-dimensional materials and techniques, while during working, process and product were discussed. Specifically, four AT interventions used only two-dimensional materials/techniques (15, 18, 21, 23), six AT interventions offered both two- and three-dimensional materials/techniques (13a, 20, 22, 29a, 30, 36), and three AT interventions offered materials/techniques fitting the topic/assignment (15, 17, 25), which included a combination of two- and three-dimensional materials/techniques. Three AT interventions let the clients work freely without topics and assignments given (13a, 18, 30), eight AT interventions were based on topics/assignments (15, 20, 21, 22, 23, 25, 29a, 36), and two AT interventions combined both ways (17, 30). Concerning the use of language, in three AT interventions, there was a discussion on process/product afterward (17, 29a, 36), in five AT interventions, there was a verbal exchange while working (13a, 15, 18, 22, 25), and five studies (16, 20, 21, 23, 30) in this category did not make their use of language explicit as an additional form of expression. The most mentioned subcategories of supposed mechanisms of change for this category were “art therapy as a form of expression to reveal what is inside,” “art therapy as a form of exploration,” and “art therapy as a way of experiencing the self.”

#### The Therapist Behavior Was Directive

The therapist showed mainly an active and leading role toward the children/adolescents; the use of means and forms of expression was again variable in this category (*n* = 10), but most often, children and adolescents worked on base of topics and assignments with both two- and three-dimensional materials/techniques, whereby the process and work were reflected upon afterward in different forms. Specifically, one intervention used only two-dimensional materials/techniques (10), five AT interventions offered both two- and three-dimensional materials/techniques (8, 11, 26, 28, 29b), and four AT interventions offered materials/techniques fitting the topic/assignment (4, 12, 13b, 24), which included two- and three-dimensional materials/techniques. Two AT interventions let the clients work without topics and assignments given (11, 28), and seven AT interventions were based on topics/assignments (4, 8, 10, 12, 13b, 24, 29b), one AT intervention combined both ways (28), and one study did not provide information on this topic (26). Concerning the use of language, in five AT interventions, there was a discussion on process/product afterward (4, 10, 11, 13b, 29b), in three AT interventions, there was a verbal exchange while working (12, 26, 28), and one study used language in a specific form (reviewing the collection with children and parents) (8). One AT intervention discussed the work afterward in a different form (a narrative retold) (4). One intervention did not make the use of language explicit as an additional form of expression (24). The most-reported subcategories of supposed mechanisms of change were the same as for the non-directive therapist behavior.

#### The Therapist Both Performed Directive and Non-directive Behavior (Eclectic) Toward Clients

Also, the use of means and forms of expression was variable in this category (*n* = 9). All kinds of materials/techniques were used but most often were worked on base of topics/assignments. The use of language was not often mentioned, but if it was used, it was used as a discussion afterward. Specifically: two AT interventions used only two-dimensional materials/techniques (7, 14), four AT interventions offered both two- and three-dimensional materials/techniques (2, 5, 32, 33), and two AT interventions offered materials/techniques fitting the topic/assignment (9, 14), which included both two- and three-dimensional materials/techniques. One study did not provide information on this topic (34). In one AT intervention, the clients worked freely without topics and assignments given (14), and six AT interventions were based on topics/assignments (2, 5, 7, 9, 32, 33). Concerning the use of language, in three AT interventions, there was a discussion on process/product afterward (2, 9, 14), no AT interventions mentioned a verbal exchange while working, and four studies (32, 33, 34, 35) in this category did not make their use of language explicit as an additional form of expression. The most-reported subcategories of supposed mechanisms of change for this category were “art therapy as a form of expression to reveal what is inside”; “art therapy as a form of exploration,” and “art therapy as an easily/safe accessible intervention.”

In seven studies (1, 3, 6, 19, 27, 31, 37), the AT interventions were not enough explicated to make combinations.

## Outcomes

### Therapist Behaviors in Relation to Psychosocial Outcomes

The division into three categories of non-directive, directive, and eclectic therapist behavior gave the opportunity to show outcomes in accordance with these. To structure the outcome, these are reported by categorizing psychosocial problems into internalizing problems, externalizing problems, and social problems and in outcomes that can be considered underlying mechanisms of psychosocial problems. These underlying mechanism outcomes were divided into the domains self-concept/self-esteem and emotion regulation.

#### Non-directive Therapist Behavior

Eight studies (15, 16, 18, 20, 21, 23, 25, 36), which applied the non-directive therapist behavior, focused on *Internalizing Problems* as an outcome. These results showed significant improvement in post-traumatic stress symptoms (23); emotional functioning (36, 16), depression, rejection, and anxiety (16), reduction of symptoms of Separation Anxiety Disorder (18), and symptoms of anxiety and depression (20). The quality of two studies (16, 23) was strong, and the other three studies were assessed as being of weak quality. Also, four times no significant improvement was reported for negative mood states (15), negative mood and distress (25), feelings of anxiety (23), and anxiety, depression, internalizing problems, and emotional symptoms (21). The quality of these studies was strong (23) or weak (15, 21, 25).

Five studies (13a, 16, 21, 29, 36) showed results for *Externalizing Problems*. The results showed significant improvement in inattention/hyperactivity problems for the Honors track group (21) (weak), behavioral conduct (29) (moderate), attention span (16) (strong), and problem behavior (13a) (moderate). However, also, no significant improvement was reported on behavioral problems (36, 16) and inattention/hyperactivity for the Average track group (21).

Four studies (16, 21, 29a, 36) reported results for *Social Problems*. A significant effect was found on social functioning and resilience (36) (weak), social acceptance (29a) (moderate), personal adjustment (21), and degree of perceived support available from others and reliance upon others (16) (strong). No significant improvement was found for personal adjustment (21). The qualitative data revealed improvement in behavioral and peer interaction (36, 21).

Some studies evaluating interventions with non-directive therapist behavior showed results on outcomes that can be considered underlying mechanisms of psychosocial problems. For the domain *Self-concept/Self-esteem*, nine studies (13a, 16, 20, 21, 23, 25, 29a, 30, 36) showed results on this domain. They reported significant improvement in self-esteem (21, 30); feelings around body image (30) (weak); self-approval (29a); sense of identity, overall personality, positive feelings about themselves (16); and resilience (36). Also, no significant improvement was shown on this domain, e.g., self-esteem (10, 21, 25, 29a), self-concept (23) (strong), and Locus of Control (13a) (refers to how strongly people believe they have control over the situations and experiences), which was a study of moderate quality. Qualitative results showed improvement in this domain on resilience (13, 36). Two studies reported results on *Emotion Regulation*. In one study, a significant improvement was seen in emotion regulation and maladaptive strategies (22) (moderate), while in another study, no improvement was found. This study was assessed as being a weak study (17). Qualitative results showed that participants reported that “ventilation of uncomfortable feelings occurred, and an outlet for alleviating stress was provided” (21), and there were improvements in emotional expression and cognition (36).

#### Directive Therapist Behavior

Four studies that applied the directive therapist behavior (4, 8, 24, 28) showed results for *Internalizing Problems*. In these studies, there was a significant improvement in internalizing behaviors (28), PTSD, and sleep-related problems (8). The quality of these studies was moderate (28) and strong (8). No significant improvement was reported for mood, depression (24), PTSD, and acute stress (4). The quality of these two studies was strong and moderate.

Four studies (10, 13b, 26, 28) reported results for *Externalizing Problems*, and significant improvement was found on anger (10), problem behavior (13b), hyperactivity/inattention (26), hyperactivity scores, and problem behavior (28). Also, no significant improvement was reported, specifically on problem behaviors (26). The qualitative results of these AT interventions described improved classroom behavior (13). The quality of these studies was moderate (10, 13, 28) and weak (26).

Four studies (11, 26, 28, 29) reported results for *Social Problems*. These studies reported significant improvement for close friendship (29) and assertion (28). But in other studies, no significant improvement was reported for social skills (26), socially lonely (11), and responsibility (28). The quality of the studies was assessed as being moderate (11, 29) and weak (26, 28). Qualitative results revealed that “the clients appeared to initiate social exchanges more independently and were improved on sharing feelings, thoughts, and ideas” (26).

Some studies applying directive therapist behavior showed results on (supposed) underlying mechanisms. Five studies (10, 11, 13, 24, 29) showed results on *Self-esteem/Self-concept*. Significant improvement was found on self-esteem (10) and self-approval (29). Also, no significant improvement was found on self-esteem (29, 24, 11), a sense of empowerment (11), responsibility for success/failure at school (11), Locus of Control (13), and educational self-esteem (10). The quality of the studies was strong (24) and moderate (10, 11, 13, 29). Also, positive qualitative results were reported in this domain, i.e., “a shift in self-image, were more confident and assured of their skills, and were more capable of expressing their ideas, thoughts, and feelings and in sharing these. They also showed an increased capacity to reflect on their behaviors and display self-awareness” (26) and improved Locus of Control (13). One study reported no significant improvement in *Emotion Regulation* (12). This study was of moderate quality.

#### Eclectic Therapist Behavior

Seven studies (2, 5, 9, 14, 32, 33, 34) in which interventions with eclectic therapist behavior was applied showed results on *Internalizing Problems*. Significant improvement was reported on internalizing problems (5), anxiety (2, 32, 33), and parent & child worry (2), depression, dissociation, sexual concerns, sexual preoccupation, and sexual distress (33), dissociative symptomatology (32), and post-traumatic stress (9, 32, 33). However, no significant results were reported on anxiety (34), depression (2, 32), dissociation (fantasy) (33), sexual concerns (32), and PTSD symptoms (14). The quality was assessed as being weak (2, 9) and moderate (14, 32, 33, 34).

Five studies (2, 5, 32, 33, 35) reported on *Externalizing Problems*, and they reported significant improvement on externalizing problems (5), problematic behaviors (35), and anger (33). No significant improvement was reported for disruptive behavior (2), hyper-response (33), and anger (2, 32). The study quality was weak (2, 35) and moderate (5, 32, 33).

Two studies (2, 7) reported on *Social Problems*, and significant improvement was found for parent and child communication (2) (weak). No significant improvement was reported on sociability, responsibility, and assertiveness (7).

Within the category eclectic therapist behavior, one study showed results on underlying mechanisms, specifically no significant improvement on *Self-concept* (34). This study was being assessed with moderate quality.

### Overall Results

As is shown in Table 3, more than 50% of the studies on the effects of AT interventions using non-directive therapist behavior showed significant effects on the outcome domains, with high impact on externalizing (80%), social problems (75%), and internalizing problems (62,5%). Self-esteem/self-concept and emotion regulation showed lower figures, with 55.6 and 50%, respectively. AT interventions in which directive therapist behavior was used showed a different picture. The number for treating externalizing problems stood out, with 100% of the studied AT interventions being significantly effective in this domain. However, percentages of significant interventions for internalizing problems, social problems, self-esteem/self-concept were equal to or <50%. AT interventions using eclectic therapist behavior showed best results on internalizing and externalizing problems with, respectively 71.4 and 60% of the AT interventions that were evaluated on these outcome domains.

**Table 3:** Number and percentage of interventions per type of therapist behavior showing significant effects on outcomes.

	**Interventions with significant effects on**				
	**Internalizing problems**	**Externalizing problems**	**Social problems**	**Self-esteem/Self-concept**	**Emotion regulation**
**THERAPEUTIC BEHAVIOR**					
Non-directive	*n* = 5 (62.5%)	*n* = 4 (80%)	*n* = 3 (75%)	*n* = 5 (55.6%)	*n* = 1 (50%)
Directive	*n* = 2 (50%)	*n* = 4 (100%)	*n* = 2 (50%)	*n* = 2 (40%)	-
Eclectic	*n* = 5 (71.4%)	*n* = 3 (60%)	*n* = 1 (50%)	-	-

## Discussion

The purpose of this systematic narrative review was to provide an overview of AT interventions that were effective in reducing psychosocial problems in children and adolescents. The emphasis was on the applied means and forms of expression during AT, the therapeutic behavior applied, and the supposed mechanisms of change to substantiate the use of the intervention. The main results showed that a broad spectrum of art materials and techniques are used in AT treatments for psychosocial problems in children and adolescents. No specific art materials or techniques stood out. Also, forms of structure such as working on the basis of topics or assignments and the way language is applied during or after the sessions vary widely and do not seem to relate to a specific category of therapist behavior. From this point of view, it seems less important which (combination of) materials/techniques and forms of structure art therapists use in treatments of psychosocial problems. The wide variety of materials, techniques, and assignments that are used in AT shows that AT is very responsive to individual cases in their treatments. This is in line with the concept that art therapists can attune to the client's possibilities and needs with art materials/techniques (Franklin, 2010).

Therapist behavior appeared to be the only distinctive component in the interventions. Three broad forms were found: non-directive, directive, and eclectic. In practice, art therapists often define their practice with orientations such as psychodynamic, gestalt, person-centered, etc. or choose an approach according to their individual preferences (Van Lith, 2016). For instance, a stance in which the therapist sees its role as being a witness to the experience of the inherent process of knowing the self (Allen, 2008) is often related to a non-directive therapist behavior or a stance in which they elicit meaning-making by engendering a new perspective (Karkou and Sanderson, 2006) is often related to a form of directive therapist behavior. Also, many art therapists work from the point of view that the art therapist should adapt to the client needs, which can be considered an eclectic approach (Van Lith, 2016) and which incorporates both forms of therapist behavior. Next to individual preferences, many psychotherapeutic approaches are being used in art therapeutic treatments of children and adolescents (Graves-Alcorn and Green, 2014; Frey, 2015; Gardner, 2015; Van Lith, 2016). However, in the end, they all range on a continuum from non-directive to directive therapist behavior (Yasenik and Gardner, 2012).

The results of this review show that AT for children and adolescents with psychosocial problems can lead to improvement in all domains for all three forms of therapist behavior in combination with a variety of means and forms. And, although the focus of this review was less on therapy outcomes, the results confirm the conclusion of Cohen-Yatziv and Regev (2019) that AT for children and adolescents with psychosocial problems can be effective. Non-directive therapist behavior, whereby the therapist is following and facilitating, shows the most significant effects in this study for psychosocial problems, next to eclectic therapist behavior for internalizing and externalizing problems. Also, it was striking that directive therapist behavior was effective for externalizing problems in all studies evaluating interventions with this type of therapist behavior, while this was not the case for the other outcome domains. Children and adolescents with externalizing problems may thus profit from directive, non-directive, and eclectic art therapist behavior. In addition, the findings suggest that we need to carefully consider using directive behavior in children with internalizing or social problems.

To substantiate the use of the AT interventions and the results, a variety of supposed mechanisms of change were described. Both specific and more general mechanisms of change were reported to substantiate AT interventions. The majority concerned specific AT mechanisms of change. Often, AT is considered a form of expression to reveal what is inside or its effects are explained by an exploration of feelings, emotions, and thoughts. These mechanisms of change were seen in AT interventions with non-directive, directive, and eclectic therapist behavior. The simultaneous occurrence of supposed mechanisms of change in all these categories of therapist behavior that differ substantially from one another can be explained by the central use of art materials, which distinguishes AT from the other ATs and from other psychotherapeutic approaches (Malchiodi, 2012). It can be considered as an additional and specific value of AT and, therefore, frequently used as substantiation for the used AT interventions and their effects.

Corresponding between the studies that showed positive results was the adaptation of the materials/techniques, forms of structure, and therapist behavior to the problems and needs of the children and adolescents involved. This process is called responsiveness. Responsiveness consists of interacting in a way such that the other is understood, valued, and supported in fulfilling important personal needs and goals. It can be seen as a moment-by-moment process of the therapeutic alliance between therapist and client (Sousa et al., 2011). Responsiveness supports and strengthens both the relationship and its members (Reis and Clark, 2013). In AT, therapist behavior and the use of materials and techniques can both be adapted to these needs and may be considered an important element in explaining the positive effects of AT. Processes such as responsiveness and therapeutic alliance relate partially to attachment theories. In AT, a therapeutic alliance includes, next to the client and art therapist, a third “object,” the art medium, comprised of art materials, art-making, and artworks (Bat Or, and Zilcha-Mano, 2018). From the perspective of attachment theory, the encounter between client and art material in AT may reflect attachment-related dynamics (Snir et al., 2017). Therefore, art therapists recapitulate positive relational aspects through purposeful creative experiences that offer sensory opportunities to reinforce a secure attachment (Malchiodi and Crenshaw, 2015). In this way, materials and techniques can offer the child and adolescent a “safe bridge” to bond with the therapist and explore and grow in developmental areas that are treated.

Given the results, relational, experiential (combined with art) knowledge to connect to the children's and adolescent's problems and needs seems indispensable for art therapists. This study included AT interventions performed by certified art therapists. Art therapists get a thorough education in relational and experiential (art) skills and obtain tacit knowledge through practice. By having more insight into the importance of the role of therapist behavior and the use of materials/techniques in AT interventions for children and adolescents, art therapists can improve results. Choices for therapist behavior and the use of materials/techniques should not depend that much on context or individual preference but on the client's problems and needs and which therapist behavior fits the client best. The results of this study provide clues on which and how to use AT elements in clinical practice, but above all, it gives a sound base for initiating more empirical research on AT. For practice and research purposes, a thorough elaboration and description of the therapist behavior in manuals are then of importance.

### Strengths and Limitations of This Review

In this study, a narrative synthesis was performed because of the focus on substantive aspects and the heterogeneity of the studies. A common criticism of narrative synthesis is that it is difficult to maintain transparency in the interpretation of the data and the development of conclusions. It threatens the value of the synthesis and the extent to which the conclusions are reliable. For instance, in this study, we searched for similarities and differences in two core elements of AT (Schweizer et al., 2014). Sometimes, forced choices had to be made in the division of the defined components into group categories and, eventually, to divide them into categories of therapist behavior. Separating and distinguishing components of an intervention are not straightforward.

From the literature, it is known that studies with positive results are overrepresented in the literature (Mlinarić et al., 2017). Probably also in this study, therefore, publication bias must be taken into account when interpreting the results.

Also, regarding showing significant results, some studies showed significant and no significant results in the same domain. This can cause bias, for example, considering a study to be significantly effective in internalizing problems, but in reality, the study shows significant results in anxiety, but for instance, not in depression. It should be taken into account that, in this study, only a broad overarching view is given.

In this study, we included RCTs, CCTs, and group pre–posttest designs because these three designs (in this order) can be considered to provide the most reliable evidence (Bondemark and Ruf, 2015). Questionable is whether these types of designs are the most appropriate designs for (a part of) the research question posed in this study. For detailed, more qualitative information on interventions, case studies seem very suitable. Potential advantages of a single case study are seen in the detailed description and analysis to gain a better understanding of “how” and “why” things happen (Ridder, 2017).

### Recommendations

Remarkably, seven studies did not describe their AT interventions sufficiently explicitly concerning the use of means and forms of expression and therapist behavior. This, while art materials/techniques and therapist behavior constitute the basis for AT interventions (Moon, 2012). Insight into the core elements of interventions helps us better understand why and how certain interventions work. By understanding these components of an intervention, we can compare interventions and improve the effectiveness of interventions (Blase and Fixsen, 2013). Therefore, for future AT studies, it is recommended to present more information on used therapeutic perspectives, means, art materials and techniques, and therapist behavior.

The results of this study show that AT interventions for children and adolescents are characterized by a variety of materials/techniques, forms of structure such as giving topics or assignments, the use of language, and therapist behavior. These results point out to more specific aspects of the dual relationship of material–therapist, which contributes to the effects, such as, for instance, responsiveness. More (qualitative) research into these specific aspects of the therapeutic relationship and the role of the relational aspects of the material could provide more insight and be of great value regarding AT for children and adolescents.

The results of the AT interventions show that AT leads to positive results for psychosocial problems, although, in some studies, both significant and not significant results were seen within a domain. A more personalized research approach, which is linked to individual treatment goals, can possibly give more clarity on the effects. Goal Attainment Scales (GAS) can be considered useful for this purpose.

## Conclusions

This study shows that the use of means and forms of expression and therapist behavior is applied flexibly. This suggests a responsiveness of AT, in which means and forms of expression and therapist behavior are applied to respond to the client's needs and circumstances, thereby giving positive (significant) results for psychosocial problems. Searching for specific elements in the use of materials and the three defined forms of therapist behavior that influence the result is therefore recommended.

## Data Availability Statement

The raw data supporting the conclusions of this article will be made available by the authors, without undue reservation.

## Author Contributions

All authors listed have made a substantial, direct and intellectual contribution to the work, and approved it for publication.

## Conflict of Interest

The authors declare that the research was conducted in the absence of any commercial or financial relationships that could be construed as a potential conflict of interest.

## References

[B1] Allen P. B. (2008). Commentary on community-based art studios: underlying principles. Art Therapy 25, 11–12. 10.1080/07421656.2008.10129350

[B2] American Art Therapy Association (2017). Facts, figures and helpful resources from AATA. Avaliable online at: https://multibriefs.com/briefs/aata/review122117.pdf (accessed July 11, 2020).

[B3] Arango C. Díaz-Caneja C. M. McGorry P. D. Rapoport J. Sommer I. E. Vorstman J. A.. (2018). Preventive strategies for mental health. Lancet Psychiatry 5, 591–604. 10.1016/S2215-0366(18)30057-9 29773478

[B4] Bat Or M. Zilcha-Mano S (2018). The Art Therapy Working Alliance Inventory: the development of a measure. International Journal of Art Therapy. 24(2), 76–87. 10.1080/17454832.2018.1518989

[B5] Baumeister R. F. Campbell J. D. Krueger J. I. Vohs K. D. (2003). Does high self-esteem cause better performance, interpersonal success, happiness, or healthier lifestyles? Psychol. Sci. Public Interest 4, 1–44. 10.1111/1529-1006.01431 26151640

[B6] Bazargan Y. Pakdaman S. (2016). The effectiveness of art therapy on reducing internalizing and externalizing problems of female adolescents. Arch. Iran. Med. 19, 51–56 26702749

[B7] Beebe A. Gelfand E. W. Bender B. (2010). A randomized trial to test the effectiveness of art therapy for children with asthma. J. Allergy Clin. Immunol. 126, 263–266.e1. 10.1016/j.jaci.2010.03.019 20462632

[B8] Beh-Pajooh A. Abdollahi A. Hosseinian S. (2018). The effectiveness of painting therapy program for the treatment of externalizing behaviors in children with intellectual disability. Vulnerable Child. Youth Stud. 13, 221–227 10.1080/17450128.2018.1428779

[B9] Bhosale S. A. Singru S. Khismatrao D. (2015). Study of Psychosocial Problems among adolescent students in Pune, India. Ameen J. Med. Sci. 8, 150–155.

[B10] Blase K. Fixsen D. (2013). Core intervention components: Identifying and operationalizing what makes programs work. Washington, DC: Office of the Assistant Secretary for Planning and Evaluation, Office of Human Services Policy, U.S. Department of Health and Human Services.

[B11] Bondemark L. Ruf S. (2015). Randomized controlled trial: the gold standard or an unobtainable fallacy? Eur. J. Orthodont. 37, 457–461. 10.1093/ejo/cjv046 26136438

[B12] Boursnell M. (2011). Parents with mental illness: the cycle of intergenerational mental illness. Child. Australia 36, 26–35. 10.1375/jcas.36.1.26

[B13] Brumariu L. E. (2015). Parent-child attachment and emotion regulation. New Dir. Child Adolesc. Dev. 2015, 31–45. 10.1002/cad.20098 26086126

[B14] Carolan R. Backos A. (2017). Emerging Perspectives in Art Therapy: Trends, Movements, and Developments, 1st Edn. New York, NY: Routledge.

[B15] Chapman L. Morabito D. Ladakakos C. Schreier H. Knudson M. M. (2001). The effectiveness of art therapy interventions in reducing Post Traumatic Stress Disorder (PTSD) symptoms in pediatric trauma patients. Art Therapy 18, 100–104. 10.1080/07421656.2001.10129750

[B16] Chaves E. (2011). The creation of art books with adolescents diagnosed with an eating disorder: effectiveness, self-esteem, and related factors (Dissertation). Retrieved from: https://digitalcommons.du.edu/etd/122/

[B17] Cho S. M. Shin Y. M. (2013). The promotion of mental health and the prevention of mental health problems in child and adolescent. Korean J. Pediatr. 56:459. 10.3345/kjp.2013.56.11.459 24348657 PMC3859877

[B18] Cohen-Yatziv L. Regev D. (2019). The effectiveness and contribution of art therapy work with children in 2018 -what progress has been made so far? A systematic review. Int. J. Art Therapy 24, 100–112. 10.1080/17454832.2019.1574845

[B19] Cuijpers P. Reijnders M. Huibers M. J. H. (2019). The role of common factors in psychotherapy outcomes. Annu. Rev. Clin. Psychol. 15, 207–231. 10.1146/annurev-clinpsy-050718-095424 30550721

[B20] Daley J. Lecroy C. W. (2001). Empowering Adolescent Girls: Examining the Present and Building Skills for the Future with the “Go Girls” Program (Norton Professional Books (Paperback)). New York, NY: W. W. Norton & Company.

[B21] D'Amico M. Lalonde C. (2017). The effectiveness of art therapy for teaching social skills to children with autism spectrum disorder. Art Therapy 34, 176–182. 10.1080/07421656.2017.1384678

[B22] Devidas N. S. A. P. Mendonca T. L. (2017). A study to evaluate the effectiveness of art therapy on self esteem among the orphans in selected orphanages at Mangalore. Asian J. Nurs. Educ. Res. 7:376 10.5958/2349-2996.2017.00075.1

[B23] Duschinsky R. Greco M. Solomon J. (2015). The politics of attachment: lines of flight with bowlby, deleuze and guattari. *Theory*. Cult. Soc. 32, 173–195. 10.1177/0263276415605577 27110049 PMC4820003

[B24] Dye M. (2018). Evaluating the benefits of art therapy interventions with grieving children (Ph. D. Thesis). James Madison University, JMU Scholarly Commons. Avaliable online at: https://commons.lib.jmu.edu/edspec201019/129

[B25] Epp K. M. (2008). Outcome-based evaluation of a social skills program using art therapy and group therapy for children on the autism spectrum. Child. Sch. 30, 27–36. 10.1093/cs/30.1.27

[B26] Erlingsson C. Brysiewicz P. (2017). A hands-on guide to doing content analysis. Afr. J. Emerg. Med. 7, 93–99. 10.1016/j.afjem.2017.08.001 30456117 PMC6234169

[B27] Fenn K. Byrne M. (2013). The key principles of cognitive-behavioral therapy. Innovation: Educ. Insp. Genrl. Pract. 6, 579–585. 10.1177/1755738012471029

[B28] Fixsen D. L. Naoom S. F. Blase K. A. Friedman R. M. Wallace F. (2005). Implementation research: a synthesis of the literature. Tampa, FL: University of South Florida, Louis de la Parte Florida Mental Health Institute, National Implementation Research Network. FMHI Publication No. 231.

[B29] Franklin M. (2010). Affect regulation, mirror neurons, and the third hand: formulating mindful empathic art interventions. Art Therapy 27, 160–167. 10.1080/07421656.2010.10129385

[B30] Freilich R. Shechtman Z. (2010). The contribution of art therapy to the social, emotional, and academic adjustment of children with learning disabilities. Arts Psychother. 37, 97–105. 10.1016/j.aip.2010.02.003

[B31] Frey D. (2015). Play therapy interventions with adults, in Play Therapy; A Comprehensive Guide to Theory and Practice, eds Crenshaw D. Stewart A. (New York, NY: Guilford), 452–464.

[B32] Gardner B. (2015). Play therapy with adolescents, in Play Therapy: A Comprehensive Guide to Theory and Practice, eds Crenshaw D. Stewart A. (New York, NY: Guilford), 439–451.

[B33] Gerge A. Hawes J. Eklöf L. Pedersen I. N. (2019). Proposed mechanisms of change in arts-based psychotherapies. Voices 19:31 10.15845/voices.v19i2.2564

[B34] Gerge A. Pedersen I. N. (2017). Analyzing pictorial artifacts from psychotherapy and art therapy when overcoming stress and trauma. The Arts in Psychotherapy. 54, 56–68. 10.1016/j.aip.2017.02.001

[B35] Gratz K. L. Levy R. Tull M. T. (2012). Emotion regulation as a mechanism of change in an acceptance-based emotion regulation group therapy for deliberate self-harm among women with borderline personality pathology. J. Cogn. Psychother. 26, 365–380. 10.1891/0889-8391.26.4.365

[B36] Graves-Alcorn S. Green E. (2014). The expressive arts therapy continuum: history and theory, in Integrating Expressive Arts and Play Therapy with Children and Adolescents, eds Green E. Drewes A. (New York, NY: Wiley), 1016.

[B37] Groh A. M. Fearon R. M. P. van IJzendoorn M. H. Bakermans-Kranenburg M. J. Roisman G. I. (2016). Attachment in the early life course: meta-analytic evidence for its role in socioemotional development. Child Dev. Perspect. 11, 70–76. 10.1111/cdep.12213

[B38] Haeyen S. van Hooren S. Dehue F. Hutschemaekers G. (2017). Development of an art-therapy intervention for patients with personality disorders: an intervention mapping study. Int. J. Art Therapy 23, 125–135. 10.1080/17454832.2017.1403458

[B39] Hartz L. Thick L. (2005). Art therapy strategies to raise self-esteem in female juvenile offenders: a comparison of art psychotherapy and art as therapy approaches. Art Therapy 22, 70–80. 10.1080/07421656.2005.10129440

[B40] Hashemian P. Jarahi L. (2014). Effect of painting therapy on aggression in educable intellectually disabled students. Psychology 05, 2058–2063. 10.4236/psych.2014.518208

[B41] Higenbottam W. (2004). In her image. Canad. Art Therapy Assoc. J. 17, 10–16. 10.1080/08322473.2004.11432256

[B42] Jang H. Choi S. (2012). Increasing ego-resilience using clay with low SES (Social Economic Status) adolescents in group art therapy. Arts Psychother. 39, 245–250. 10.1016/j.aip.2012.04.001

[B43] Jaspers M. de Winter A. F. Veenstra R. Ormel J. Verhulst F. C. Reijneveld S. A. (2012). Preventive child health care findings on early childhood predict peer-group social status in early adolescence. J. Adolesc. Health 51, 637–642. 10.1016/j.jadohealth.2012.03.017 23174476

[B44] Jo M. J. Hong S. Park H. R. (2018). Effects of art intervention program for siblings of children with cancer: a pilot study. J. Pediatr. Oncol. Nurs. 35, 178–187. 10.1177/1043454218762702 29577798

[B45] Karkou V. Sanderson P. (2006). Arts Therapies: a Research-Based Map of the Field. Edinburgh: Elsevier Churchill Livingstone.

[B46] Khadar M. G. Babapour J. Sabourimoghaddam H. (2013). The effect of art therapy based on painting therapy in reducing symptoms of Oppositional Defiant Disorder (ODD) in elementary school boys. Procedia Soc. Behav. Sci. 84, 1872–1878. 10.1016/j.sbspro.2013.07.051

[B47] Khodabakhshi Koolaee A. Vazifehdar R. Bahari F. Akbari M. E. (2016). Impact of painting therapy on aggression and anxiety of children with cancer. Caspian J. Pediatr. 2, 135–141.

[B48] Kieling C. Baker-Henningham H. Belfer M. Conti G. Ertem I. Omigbodun O.. (2011). Child and adolescent mental health worldwide: evidence for action. Lancet 378, 1515–1525. 10.1016/S0140-6736(11)60827-1 22008427

[B49] Kymissis P. Christenson E. Swanson A. J. Orlowski B. (1996). Group treatment of adolescent inpatients: a pilot study using a structured therapy approach. J. Child Adolesc. Group Therapy 6, 45–52. 10.1007/BF02548513

[B50] Lehman B. J. David D. M. Gruber J. A. (2017). Rethinking the biopsychosocial model of health: understanding health as a dynamic system. Soc. Personal. Psychol. Compass 11:e12328 10.1111/spc3.12328

[B51] Liu C. (2017). Examining the effectiveness of Solution-Focused Art Therapy (SF-AT) for sleep problems of children with traumatic experience. Avaliable online at: https://www.semanticscholar.org/paper/Examining-the-Effectiveness-of-Solution-Focused-Art-Liu/07047beab98ae0d76fad50d96ebc13f0da2d6ffb

[B52] Lyshak-Stelzer F. Singer P. Patricia St. John P. Chemtob C. M. (2007). Art therapy for adolescents with posttraumatic stress disorder symptoms: a pilot study. Art Therapy 24, 163–169. 10.1080/07421656.2007.10129474

[B53] Malchiodi C. (2007). Art Therapy Sourcebook. New York, NY: McGraw-Hill Education.

[B54] Malchiodi C. A. (2012). Art Therapy and Health Care, 1st Ed. New York, NY: The Guilford Press.

[B55] Malchiodi C. A. Crenshaw D. A. (2015). Creative Arts and Play Therapy for Attachment Problems. New York, NY: The Guilford Press.

[B56] Mlinarić A. Horvat M. Šupak Smolčić V. (2017). Dealing with the positive publication bias: why you should really publish your negative results. Biochem. Medica 27, 13–15. 10.11613/BM.2017.030201 29180912 PMC5696751

[B57] Moon B. L. (2012). Art therapy teaching as performance art. Art Therapy 29, 192–195. 10.1080/07421656.2012.730954

[B58] Ogundele M. O. (2018). Behavioral and emotional disorders in childhood: a brief overview for pediatricians. World J. Clin. Pediatr. 7, 9–26. 10.5409/wjcp.v7.i1.9 29456928 PMC5803568

[B59] Patrick P. M. Reupert A. E. McLean L. A. (2019). A cross-sectional study on intergenerational parenting and attachment patterns in adult children of parents with mental illness. Child Fam. Soc. Work 24, 601–609. 10.1111/cfs.12641

[B60] Petri G. Beadle-Brown J. Bradshaw J. (2020). Redefining self-advocacy: a practice theory-based approach. J. Policy Pract. Intel. Disabilities 17, 207–218. 10.1111/jppi.12343

[B61] Pifalo T. (2002). Pulling out the thorns: art therapy with sexually abused children and adolescents. Art Therapy 19, 12–22. 10.1080/07421656.2002.10129724

[B62] Pifalo T. (2006). Art therapy with sexually abused children and adolescents: extended research study. Art Therapy 23, 181–185. 10.1080/07421656.2006.10129337

[B63] Pretorius G. Pfeifer N. (2010). Group art therapy with sexually abused girls. S. Afr. J. Psychol. 40, 63–73. 10.1177/008124631004000107

[B64] Ramin A. Mousavi M. Sohrabi N. (2014). Effects of art therapy on anger and self-esteem in aggressive children. Procedia Soc. Behav. Sci. 113, 111–117. 10.1016/j.sbspro.2014.01.016

[B65] Ramirez K. A. (2013). Art therapy for enhancing academic experience of male high school freshmen (Ph. D. Dissertation). Avaliable online at: https://digitalcommons.lesley.edu/expressive_dissertations/28/?utm_source=digitalcommons.lesley.edu%2Fexpressive_dissertations%2F28&utm_medium=PDF&utm_campaign=PDFCoverPage/

[B66] Regev D. Guttmann J. (2005). The psychological benefits of artwork: the case of children with learning disorders. Arts Psychother. 32, 302–312. 10.1016/j.aip.2005.02.001

[B67] Reis H. T. Clark M. S. (2013). Responsiveness, in Oxford Library of Psychology. The Oxford Handbook of Close Relationships, eds Simpson J. A. Campbell L. (Oxford University Press), 400–423.

[B68] Richard D. A. More W. Joy S. P. (2015). Recognizing emotions: testing an intervention for children with autism spectrum disorders. Art Therapy 32, 13–19. 10.1080/07421656.2014.994163

[B69] Ridder H. G. (2017). The theory contribution of case study research designs. Bus. Res. 10, 281–305. 10.1007/s40685-017-0045-z

[B70] Riley S. (1999). Brief therapy: an adolescent invention. Art Therapy 16, 83–86. 10.1080/07421656.1999.10129669

[B71] Rosal M. L. (1993). Comparative group art therapy research to evaluate changes in locus of control in behavior disordered children. Arts Psychother. 20, 231–241. 10.1016/0197-4556(93)90018-W

[B72] Ross C. (1996). Something to Draw On: Activities and Interventions Using an Art Therapy Approach. 1st Edn London, UK: Jessica Kingsley.

[B73] Rowe C. Watson-Ormond R. English L. Rubesin H. Marshall A. Linton K.. (2016). Evaluating art therapy to heal the effects of trauma among refugee youth. Health Promot. Pract. 18, 26–33. 10.1177/1524839915626413 26933006

[B74] Ruiz M. (1997). The Four Agreements. San Rafael, CA: Amber-Allen Pub.

[B75] Saunders E. J. Saunders J. A. (2000). Evaluating the effectiveness of art therapy through a quantitative, outcomes-focused study. Arts Psychother. 27, 99–106. 10.1016/S0197-4556(99)00041-6

[B76] Scheeringa M. S. Salloum A. Arnberger R. A. Weems C. F. Amaya-Jackson L. Cohen J. A. (2007). Feasibility and effectiveness of cognitive-behavioral therapy for posttraumatic stress disorder in preschool children: two case reports. J. Trauma. Stress 20, 631–636. 10.1002/jts.20232 17721975 PMC7023908

[B77] Schreier H. Ladakakos C. Morabito D. Chapman L. Knudson M. M. (2005). Posttraumatic stress symptoms in children after mild to moderate pediatric trauma: a longitudinal examination of symptom prevalence, correlates, and parent-child symptom reporting. J. Trauma. 58, 353–363. 10.1097/01.TA.0000152537.15672.B7 15706200

[B78] Schweizer C. Knorth E. J. Spreen M. (2014). Art therapy with children with Autism spectrum disorders: a review of clinical case descriptions on ‘what works.’ Arts Psychother. 41, 577–593. 10.1016/j.aip.2014.10.009

[B79] Sellers R. Collishaw S. Rice F. Thapar A. K. Potter R. Mars B.. (2013). Risk of psychopathology in adolescent offspring of mothers with psychopathology and recurrent depression. Br. J. Psychiatry 202, 108–114. 10.1192/bjp.bp.111.104984 23060622

[B80] Siegel J. Iida H. Rachlin K. Yount G. (2016). Expressive arts therapy with hospitalized children: a pilot study of co-creating healing sock creatures©. J. Pediatr. Nurs. 31, 92–98. 10.1016/j.pedn.2015.08.006 26382965

[B81] Sitzer D. L. Stockwell A. B. (2015). The art of wellness: a 14-week art therapy program for at-risk youth. Arts Psychother. 45, 69–81. 10.1016/j.aip.2015.05.007

[B82] Smith J. P. Smith G. C. (2010). Long-term economic costs of psychological problems during childhood. Soc. Sci. Med. 71, 110–115. 10.1016/j.socscimed.2010.02.046 20427110 PMC2887689

[B83] Snir S. Regev D. Shaashua Y. H. (2017). Relationships between attachment avoidance and anxiety and responses to art materials. Art Therapy 34, 20–28. 10.1080/07421656.2016.1270139

[B84] Soliman E. S. Mahdy R. S. Fouad H. A. Abbas R. A. Fayed A. (2020). Multiple risk factors affecting childhood psychosocial dysfunction in primary school Egyptian children. Middle East Current Psychiatry 27:16 10.1186/s43045-020-00023-2

[B85] Sousa Z. Ribeiro E. Horvath A. (2011). Therapeutic responsiveness as a moment-to-moment process of alliance: development of a conceptual-empirical model and an observational system (Ph. D. Thesis). Avaliable online at: https://www.researchgate.net/publication/289374352_Therapeutic_Responsiveness_as_a_moment-to-moment_process_of_Alliance_Development_of_a_conceptual-empirical_model_and_an_observational_system (accessed July 16, 2020).

[B86] Stafstrom C. E. Havlena J. Krezinski A. J. (2012). Art therapy focus groups for children and adolescents with epilepsy. Epilepsy Behav. 24, 227–233. 10.1016/j.yebeh.2012.03.030 22554978

[B87] Steiert A. (2015). Helden(–bilder) als emotionale unterstützung in der kunsttherapie mit kindern und jugendlichen im kontext lebensbedrohlicher erkrankungen. Musik Tanz-Und Kunsttherapie 26, 24–28. 10.1026/0933-6885/a000159

[B88] Teel S. (2007). Defending and Parenting Children Who Learn Differently. Santa Barbara: Praeger.

[B89] Thomas B. H. Ciliska D. Dobbins M. Micucci S. (2004). A process for systematically reviewing the literature: providing the research evidence for public health nursing interventions. Worldviews Evid. Based Nurs. 1, 176–184. 10.1111/j.1524-475X.2004.04006.x 17163895

[B90] Tibbetts T. J. Stone B. (1990). Short-term art therapy with seriously emotionally disturbed adolescents. Arts Psychother. 17, 139–146. 10.1016/0197-4556(90)90024-K

[B91] Van Lith T. (2016). Art therapy in mental health: a systematic review of approaches and practices. Arts Psychother. 47, 9–22. 10.1016/j.aip.2015.09.003

[B92] Veldman K. Reijneveld S. A. Almansa Ortiz J. Verhulst F. C. Bültmann U. (2015). Mental health trajectories from childhood to young adulthood affect the educational and employment status of young adults: results from the TRAILS study. J. Epidemiol. Commun. Health 69, 588–593. 10.1136/jech-2014-204421 25667302

[B93] Veríssimo M. Santos A. J. Fernandes C. Shin N. Vaughn B. E. (2014). Associations between attachment security and social competence in preschool children. Merrill-Palmer Q. 60:80 10.13110/merrpalmquar1982.60.1.0080

[B94] Vogels A. G. C. (2008). The Identification by Dutch Preventive Child Health Care of Children With Psychosocial Problems: Do Short Questionnaires Help? (Thesis). Faculty of Medical Sciences, University Medical Center Groningen, The Netherlands.

[B95] Wallace J. Packman W. Huffman L. C. Horn B. Cowan M. Amylon M. D. (2014). Psychosocial changes associated with participation in art therapy interventions for siblings of pediatric hematopoietic stem cell transplant patients. Art Therapy 31, 4–11. 10.1080/07421656.2014.873685

[B96] Waller D. (2006). Art therapy for children: how it leads to change. Clin. Child Psychol. Psychiatry 11, 271−282. 10.1177/1359104506061419 17086689

[B97] Walsh S. M. (1993). Future images: An art intervention with suicidal adolescents. Appl Nurs Res. 6, 111–118. 10.1016/s0897-1897(05)80171-5 8239639

[B98] World Health Organization (2018). Child and adolescent mental health. Avaliable online at: https://www.who.int/mental_health/maternal-child/child_adolescent/en/ (accessed August 11, 2020).

[B99] Wright A. G. C. Simms L. J. (2015). A metastructural model of mental disorders and pathological personality traits. Psychol. Med. 45, 2309–2319. 10.1017/S0033291715000252 25903065 PMC4498970

[B100] Yasenik L. Gardner K. (2012). Play Therapy Dimension Model: A Decision Making Guide For Integrative Play Therapists, 2nd Edn. Philadelphia, PA: Jessica Kingsley.

