# Supplementary material for: Art Therapy for Psychosocial Problems in Children and Adolescents: A Systematic Narrative Review on Art Therapeutic Means and Forms of Expression, Therapist Behavior, and Supposed Mechanisms of Change
Source: Front Psychol. 2020 Oct 8;11:584685. doi: 10.3389/fpsyg.2020.584685 (PMC7578380; doi:10.3389/fpsyg.2020.584685)
Supplement: Supplementary file 1 [file Data_Sheet_1.pdf]

Appendix 1. Searchstrategy/searchstrings

Pubmed

(("Art Therapy"[Mesh] OR Art therapy[tw] OR arts therapy[tw] OR Clay modelling[tw] OR Self-expression[tw] OR self expression[tw]) AND ("Behavioral Symptoms"[Mesh] OR "Anxiety"[Mesh:NoExp] OR "Phobia, Social"[Mesh] OR "Psychosomatic Medicine"[Mesh] OR "Psychophysiologic Disorders"[Mesh] OR "Attention"[Mesh] OR "Disruptive, Impulse Control, and Conduct Disorders"[Mesh] OR Aggress*[tw] OR Depress*[tw] OR Affect*[tw] OR Attach*[tw] OR Reaction*[tw] OR Anxiety[tw] OR Seperat*[tw] OR Phobia[tw] OR Psychosomatic[tw] OR Attent*[tw] OR Disrupt*[tw] OR Impulse control[tw] OR Conduct disorder[tw] OR Trauma[tw] OR Development disorder*[tw] OR developmental disorder*[tw] OR Emotional disorder*[tw] OR Behavior disorder*[tw] OR behavioral disorder*[tw] OR Autism[tw] OR Psychosocial adjustment[tw] OR Concentration[tw] OR Juvenile delinquency[tw]) AND ("Child"[Mesh] OR "Adolescent"[Mesh] OR Child[tw] OR Adolescen*[tw] OR Youth[tw] OR Juvenile[tw]) AND ("Randomized Controlled Trial" [Publication Type] OR Random* OR Clinical trial OR Controlled clinical trial OR Clinical study))

Medline

(('art therapy'/exp OR 'art'/exp OR 'creativity'/exp OR 'art therapy'/exp OR 'art'/exp OR 'creativity'/exp OR “Art therapy” OR “arts therapy” OR “Clay modelling” OR “Self-expression” OR “self expression”) AND ('psychological adjustment'/exp OR 'emotional attachment'/exp OR 'emotionality'/exp OR 'emotional disorder'/exp OR 'emotion'/exp OR 'emotional stability'/exp OR 'emotional stress'/exp OR 'behavior disorder'/exp 'depression'/exp OR 'anxiety'/exp OR 'psychosomatic disorder'/exp OR 'aggression'/exp OR 'behavior disorder'/exp OR 'concentration loss'/exp OR 'impulsiveness'/exp OR 'juvenile delinquency'/exp OR Aggress* OR Depress* OR Affect* OR Attach*OR Reaction* OR Anxiety OR Seperat* OR Phobia OR Psychosomatic OR Attent* OR Disrupt* OR ‘Impulse control’ OR ‘Conduct disorder’ OR Trauma OR ‘Development disorder*’ OR ‘developmental disorder*’ OR ‘Emotional disorder*’ OR ‘Behavior disorder*’ OR ‘behavioral disorder*’ OR Autism OR ‘Psychosocial adjustment’ OR Concentration OR ‘Juvenile delinquency’) AND ('child'/exp 'adolescent'/exp OR Child* OR Adolescen* OR Youth OR Juvenile) AND ('randomized controlled trial'/exp OR Random* OR ‘Clinical trial’ OR ‘Controlled clinical trial’ OR ‘Clinical study’))

Cinahl + add in advanced search: preschool, child and adolescents + exclude Medline

((MH "Art Therapy" OR “Art therapy” OR “arts therapy” OR “Clay modelling” OR “Self-expression” OR “self expression”) AND (MH "Behavioral and Mental Disorders+" OR Aggress* OR Depress* OR Affect* OR Attach* OR Reaction* OR Anxiety OR Seperat* OR Phobia OR Psychosomatic OR Attent* OR Disrupt* OR “Impulse control” OR “Conduct disorder” OR Trauma OR “Development disorder* OR “developmental disorder*” OR “Emotional disorder*” OR “Behavior disorder*” OR “behavioral disorder*” OR Autism OR “Psychosocial adjustment” OR Concentration OR “Juvenile delinquency”) AND (MH "Child" MH "Adolescence+" AND Child* OR Adolescen* OR Youth OR Juvenile) AND (MH "Clinical Trials+" OR Random* OR “Clinical trial” OR “Controlled clinical trial” OR “Clinical study”)) ((MH "Art Therapy" OR “Art therapy” OR “arts therapy” OR “Clay modelling” OR “Self-expression” OR “self expression”) AND (MH "Behavioral and Mental Disorders+" OR Aggress* OR Depress* OR Affect* OR Attach* OR Reaction* OR Anxiety OR Seperat* OR Phobia OR Psychosomatic OR Attent* OR Disrupt* OR “Impulse control”

OR “Conduct disorder” OR Trauma OR “Development disorder* OR “developmental disorder*” OR “Emotional disorder*” OR “Behavior disorder*” OR “behavioral disorder*” OR Autism OR “Psychosocial adjustment” OR Concentration OR “Juvenile delinquency”) AND (MH "Child" MH "Adolescence+" AND Child* OR Adolescen* OR Youth OR Juvenile) AND (MH "Clinical Trials+" OR Random* OR “Clinical trial” OR “Controlled clinical trial” OR “Clinical study”))

PsycInfo + add in advanced search: preschool, school age, adolescence + human + clinical trial, empirical study, literature review, meta-analysis, qualitative study, treatment outcome

((DE "Art Therapy" OR DE "Creativity" OR DE “Art” OR DE "Educational Therapy" OR DE “Self Expression” OR “Art therapy” OR “arts therapy” OR “Clay modelling” OR “Self-expression” OR “self expression”) AND (DE “Emotions” OR DE “Emotional Regulation” OR DE “Emotional Control” OR DE “Emotional Development” OR DE "Emotional Stability" OR DE “Emotional Trauma” OR DE “Empathy” OR DE “Emotional Adjustment” OR DE “Somatoform Disorders” OR DE “Psychosocial Readjustment” OR DE “Psychosocial Rehabilitation” OR DE "Anxiety" OR DE "Major Depression" OR DE “Aggressive Behavior” OR DE “Concentration” OR DE "Self-Control” OR DE “Predeliquent Youth” OR DE "Social Behavior" OR DE “Social Adjustment” OR DE "Social Learning" OR DE "Imitation (Learning)" OR DE "Imprinting" OR DE "Social Phobia" OR DE “Behavioral Inhibition” OR DE “Trauma” OR Aggress* OR Depress* OR Affect* OR Attach* OR Reaction* OR Anxiety OR Seperat* OR Phobia OR Psychosomatic OR Attent* OR Disrupt* OR “Impulse control” OR “Conduct disorder” OR Trauma OR “Development disorder* OR “developmental disorder*” OR “Emotional disorder*” OR “Behavior disorder*” OR “behavioral disorder*” OR Autism OR “Psychosocial adjustment” OR Concentration OR “Juvenile delinquency”) AND Random* OR “Clinical trial” OR “Controlled clinical trial” OR “Clinical study”))

The Cochrane Library + title, abstract, keyword

((“Art Therapy” OR “arts therapy” OR “Self-expression” OR “self expression”) AND (Child* OR Adolescen* OR Youth OR Juvenile) AND (“Randomized Controlled Trial” OR Random* OR “Clinical trial” OR “Controlled clinical trial” OR “Clinical study”))

Web of Science

((“Art Therapy” OR “arts therapy” OR “Self-expression” OR “self expression”) AND (Child* OR Adolescen* OR Youth OR Juvenile) AND (“Randomized Controlled Trial” OR Random* OR “Clinical trial” OR “Controlled clinical trial” OR “Clinical study”))

Google Scholar

“Art Therapy” OR “arts therapy” OR “Self-expression” OR “self expression” AND Children OR Adolescents OR Youth OR Juvenile AND “Randomized Controlled Trial” OR Randomized OR “Clinical trial” OR “Controlled clinical trial” OR “Clinical study” AND "psychosocial problems" OR Affective OR Attachment OR Reactions OR Anxiety OR Seperation OR Phobia OR Psychosomatic OR Attention OR Disruptive OR “Impulse control” OR “Conduct disorder” OR Trauma OR “Development disorder OR “developmental disorder” OR “Emotional disorder” OR “Behavior disorder” OR “behavioral disorder” OR Autism OR “Psychosocial adjustment” OR Concentration OR “Juvenile delinquency”

Eric

(("Art Therapy" OR "Color Therapy" OR "art therapy" OR "arts therapy" OR "art therapies" OR "arts therapies" OR "visual art therapy" OR "visual arts therapy" OR "Color Therapy" OR "Color Therapies" OR "Colour Therapy" OR "Colour Therapies" OR "painting

therapy" OR "drawing therapy" OR "sculpting therapy" OR "clay therapy" OR "clay sculpting" OR clay model*) AND (“Behavioral Symptoms” OR “Emotional Adjustment” OR “Social Problems” OR “Psychosocial Adaptation” OR “Psychophysiologic Disorders” OR “Mental Disorders” OR Behavior OR Aggress* OR Depress* OR Affect* OR Reaction* OR Anxiety OR Seperat* OR Phobia OR Psychosomatic OR Attent* OR Disrupt* OR “Impulse control” OR “Conduct disorder” OR Trauma OR “Development disorder*” OR “developmental disorder*” OR “Emotional disorder*” OR “Behavior disorder*” OR “behavioral disorder*” OR Autism OR “Psychosocial adjustment” OR Concentration OR “Juvenile delinquency”) AND (Child* OR Adolescen* OR Youth OR Juvenile) AND (“Randomized Controlled Trial” OR Random* OR “Clinical trial” OR “Controlled clinical trial” OR “Clinical study”)

Merkurstab

((“Art Therapy” OR “arts therapy” OR “Self-expression” OR “self expression”) AND (Child* OR Adolescen* OR Youth OR Juvenile) AND (“Randomized Controlled Trial” OR Random* OR “Clinical trial” OR “Controlled clinical trial” OR “Clinical study”))

Arthedata

((“Art Therapy” OR “arts therapy” OR “Self-expression” OR “self expression”) AND (Child* OR Adolescen* OR Youth OR Juvenile) AND (“Randomized Controlled Trial” OR Random* OR “Clinical trial” OR “Controlled clinical trial” OR “Clinical study”))
